# Potent modulation of the CepR quorum sensing receptor and virulence in a *Burkholderia cepacia* complex member using non-native lactone ligands

**DOI:** 10.1038/s41598-019-49693-x

**Published:** 2019-09-17

**Authors:** Betty L. Slinger, Jacqueline J. Deay, Josephine R. Chandler, Helen E. Blackwell

**Affiliations:** 1 Department of Chemistry, University of Wisconsin–Madison, 1101 University Ave., Madison, WI 53706 USA; 2 Department of Molecular Biosciences, University of Kansas, 1200 Sunnyside Ave., Lawrence, KS 66045 USA

**Keywords:** Chemical tools, Pathogens

## Abstract

The *Burkholderia cepacia* complex (Bcc) is a family of closely related bacterial pathogens that are the causative agent of deadly human infections. Virulence in Bcc species has been shown to be controlled by the CepI/CepR quorum sensing (QS) system, which is mediated by an *N*-acyl L-homoserine lactone (AHL) signal (C_8_-AHL) and its cognate LuxR-type receptor (CepR). Chemical strategies to block QS in Bcc members would represent an approach to intercept this bacterial communication process and further delineate its role in infection. In the current study, we sought to identify non-native AHLs capable of agonizing or antagonizing CepR, and thereby QS, in a Bcc member. We screened a library of AHL analogs in cell-based reporters for CepR, and identified numerous highly potent CepR agonists and antagonists. These compounds remain active in a Bcc member, *B*. *multivorans*, with one agonist 250-fold more potent than the native ligand C_8_-AHL, and can affect QS-controlled motility. Further, the CepR antagonists prolong *C*. *elegans* survival in an infection model. These AHL analogs are the first reported non-native molecules that both directly modulate CepR and impact QS-controlled phenotypes in a Bcc member, and represent valuable chemical tools to assess the role of QS in Bcc infections.

## Introduction

Members of the *Burkholderia cepacia* complex (Bcc) have emerged as multi-drug resistant opportunistic pathogens in human infections, including pulmonary infection associated with cystic fibrosis (CF)^1^ and central nervous system infections^2^. The Bcc consists of more than 17 closely-related species that are mostly found in the environment and has grown to include not only plant, animal, and human pathogens, but also bioremediation and biocontrol strains. While all Bcc members have the potential to infect CF patients^3^, the most commonly isolated species from these patients are *B*. *cenocepacia* and *B*. *multivorans*^4^. Infections are especially problematic due to these species having high levels of intrinsic and acquired resistance to many clinically-relevant antibiotics (recently reviewed in ref.^5^). Furthermore, promising new antibiotics for the Bcc, such as Q22^6^, were subsequently shown to increase resistance after treatment and had toxicity effects in the host^7^. Consequently, new therapeutic strategies are needed to treat Bcc infections.

Because pathogenicity and virulence phenotypes are under the control of quorum sensing (QS) in many common bacterial pathogens, including the Bcc, QS inhibition has attracted interest as a potential strategy to combat bacterial infections^8^. Quorum sensing (QS) is a bacterial cell-cell signaling system based on small molecule or peptide signals, termed autoinducers. Proteobacteria commonly use diffusible *N*-acyl L-homoserine lactone (AHL) autoinducers, which are generated by LuxI-type synthases and sensed by cytoplasmic LuxR-type receptors. Signal concentration increases with cell density, and when a threshold population is achieved in a given environment, the QS signal will productively bind to its target receptor protein. This binding event will ultimately cause alterations in gene expression levels, allowing the bacteria to initiate group-beneficial behaviors. QS allows pathogens to coordinate production of factors that increase infectivity (e.g., toxins, exoenzymes, and biofilm matrix components)^9^. There is evidence to suggest that targeting the QS system may put less selective pressure on these pathogens and could avoid the development of resistant bacteria^10^. Therefore, QS systems have been proposed as promising targets for the development of new anti-infective compounds^11^.

**Figure 1:**
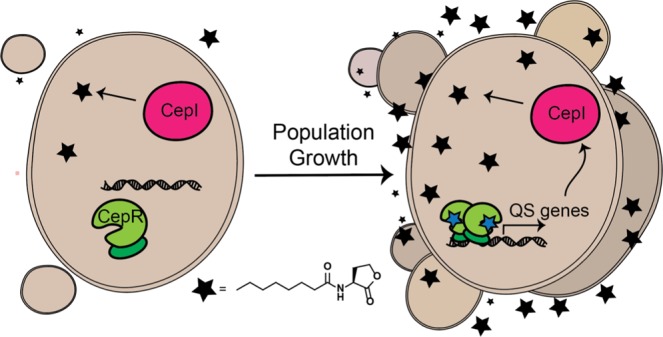
*Simplified QS circuit in Bcc bacteria*. The CepI synthase (pink) produces the diffusible *N*-octanoyl L-homoserine lactone (C_8_-AHL, black star) at a low basal level. With population growth or bacterial confinement, signal concentration will reach a threshold intracellular level upon which C_8_-AHL will bind the CepR receptor (green), potentially induces receptor homodimerization, and turns on expression of QS-controlled genes. Lighter and darker green portions of CepR indicate the putative ligand and DNA binding domains, respectively.

All of the members of the Bcc investigated so far contain the CepI/CepR QS system, which synthesizes and responds to the autoinducer *N*-octanoyl L-homoserine lactone (C_8_-AHL, Fig. 1) via the CepI synthase and CepR receptor, respectively. QS is a central regulator for the expression of pathogenic traits in the Bcc^12^, and the CepI/CepR circuit has been demonstrated to control virulence in various eukaryotic models of Bcc infection, including *C*. *elegans*^13^, fruit fly (*D*. *melanogaster*)^14^, rats, and mice^15,16^. Furthermore, a functional QS system is maintained in chronic *B*. *cenocepacia* and *B*. *multivorans* CF-infections^17^, which is intriguing because the related LasR receptor from the most commonly isolated CF-pathogen, *Pseudomonas aeruginosa*, can become non-functional during the course of chronic CF-infections^18–20^. This result underscores the likely importance of the CepI/CepR system to Burkholderia physiology and Bcc pathogenicity.

Methods to inhibit QS in the Bcc could be extremely valuable for both fundamental studies of the CepI/CepR system and for infection control. In addition, strategies that selectively target CepR in a mixed microbial setting could illuminate the role of possible interspecies interactions via QS in polymicrobial infections or environments. Small molecules that agonize or antagonize CepI/CepR would represent one such strategy. Our laboratory has developed hundreds of synthetic AHLs and non-AHL-derived compounds that can either inhibit or activate LuxR-type receptors in many different species of Gram-negative bacteria. For example, these compounds have proven successful at modulating QS in *P*. *aeruginosa* (ligands targeting LasR, RhlR, or QscR)^21,22^, *Acinetobacter baumannii* (AbaR)^23^, *Agrobacterium tumefaciens* (TraR)^24,25^, *Vibrio fischeri* (LuxR)^25^, *Chromobacterium violaceum* (CviR)^26^, *Pectobacterium carotovora* (ExpR1 and ExpR2)^27^, *Pseudomonas syringae* (AhlR)^28^, and *Rhodopseudomonas palustris* (RpaR)^29^. In the work outlined herein, we sought to apply our research strategy to identify chemical modulators of CepR and utilize these ligands to modulate QS, and thereby virulence, in a member of the Bcc.

To date, there has been limited research focused on developing compounds that target the CepI/CepR system, or modulate QS in general, in Bcc member species. In fact, we are only aware of five reported studies^30–34^. An early report by Weingart *et al*. provided some clues as to which structural features of naturally-occurring AHLs may affect CepR function^30^, and identified hexanoyl-HL and 5-hexynoyl-HL as having slight inhibitory effects on CepR at concentrations greater than 1 μM, although potency data (i.e., EC_50_ and IC_50_ values) were not reported. More recently, Scoffone and coworkers reported several diketopiperazine derivatives were CepI synthase inhibitors and increased survival of *B*. *cenocepacia*-infected *Caenorhabditis elegans*^35^. Other compounds have been shown to affect QS-controlled phenotypes in certain Bcc members. For example, Riedel and coworkers demonstrated that a hydrazide type compound (termed compound 3), could alter QS-controlled phenotypes in *B*. *cenocepacia* H111^33^. In addition, Brackman and colleagues discovered that two different compounds, baicalin hydrate^32^ and a triazole compound^34^, can reduce biofilm biomass (a QS-controlled phenotype) in *B*. *cenocepacia* and *B*. *multivorans*. However, in all of these studies, the molecular target of the compounds was not demonstrated, and it was subsequently discovered that baicalin hydrate likely targets bacterial respiration (not QS)^36^. These prior studies indicate that additional research is required to identify chemical tools to selectively target CepR.

Herein, we report the systematic screening of an AHL-based library in an *E*. *coli* CepR reporter strain and the discovery of a set of highly potent CepR modulators. The CepR antagonists have sub- to single-digit micromolar IC_50_ values and many of the CepR agonists are more potent than CepR’s native ligand, C_8_-AHL, with EC_50_ values below 1 nM in the reporter strain. These compounds remain active in a member of the Bcc, *B*. *multivorans*, and affect QS-controlled motility, with antagonists reducing motility by roughly 50%. Notably, our lead CepR antagonists increase the survival of *C*. *elegans* colonized by *B*. *multivorans*, suggesting that *B*. *multivorans* pathogenicity was attenuated by targeting CepR and inhibiting QS-controlled pathways. Scrutiny of the lead ligands allowed us to develop the first structure-activity relationships (SARs) for AHL-mediated CepR antagonism and agonism, which will provide a launching point for the design of yet more potent and potentially receptor selective ligands for CepR. These compounds represent valuable new chemical tools to study the role of CepR and QS in the virulence of Bcc members.

## Results

### Selection of compounds for testing

Our laboratory has previously reported the design, synthesis, and evaluation of several libraries of native AHLs, non-native AHL, and AHL-like compounds^22,25,26,37^. We turned to these in-house libraries to identify modulators of CepR in the Bcc (see Fig. S1 for structures of the selected 169 compounds). The majority of these compounds contained a lactone “head group” with a variety of acyl “tail groups” that could presumably occupy the same space as C_8_-AHL in the CepR ligand-binding site, and thereby act as competitive agonists or antagonists.

### Development of an *E*. *coli* β-galactosidase reporter for CepR

We began our study by screening the AHL library for CepR modulators using an *E*. *coli*-based two plasmid reporter system (Ec-reporter)^38–40^. Certain members of the Bcc harbor additional QS systems that are either AHL-based (e.g., BviI/BviR in *B*. *vietnamiensis*)^41^ or diffusible signal factor (DSF)-based (*B*. *cenocepacia*)^42^, and at least one member of the Bcc has multiple receptors that respond to C_8_-AHL (e.g., CepR and CepR2 in *B*. *cenocepacia*)^43^. Thus, we reasoned that using a CepR reporter in a heterologous background could simplify our initial analyses. We modified our previously reported *E*. *coli*-based two plasmid β-galactosidase reporter system to develop a CepR Ec-reporter (see Methods). The first plasmid, pJN105-*cepR*, contains CepR from *B*. *cenocepacia* under the control of the L-arabinose inducible pBAD promoter. A second plasmid contains a transcriptional fusion of *lacZ* to the *cepI* promoter region. The two plasmids were co-transformed into a QS mutant *E*. *coli* strain (JLD271 ∆*sdiA*) that lacks its LuxR-type receptor, SdiA. Using this reporter system, if an exogenously -added compound is able to interact with CepR, this will affect the protein’s interaction with the *cepI* promoter region and alter β-galactosidase production. Enzyme production, and thereby CepR activity, is then measurable via the degradation of a colorimetric substrate for β-galactosidase. Using this new Ec-reporter, we observed robust activity of CepR in response to its native ligand C_8_-AHL, with an EC_50_ of 5 nM (Table 1).

**Table 1:** CepR agonism data for selected compounds in the *E*. *coli* reporter^a^.

Compound	Maximal Activation (%)	EC_50_ (nM)	95% CI (nM)^b^
C_4_-AHL	23	—	—
C_6_-AHL	87	≥20000^c^	—
C_8_-AHL	100	5.7	4.6–6.9
3’OH-C_8_-AHL	84	210	160–280
3’O-C_8_-AHL	86	1200	720–2500
D-3’O-C_8_-AHL	85	≥27000^c^	—
C_10_-AHL	87	110	83–140
C_12_-AHL	98	310	180–520
C_14_-AHL	49	—	—
C_16_-AHL	35	—	—
**A11**	−5.0	—	—
**A12**	46	—	—
**A13**	110	150	100–240
**B7**	97	0.29	0.14–0.59
**B9**	88	1000	590–2100
**B11**	87	130	85–200
**E26**	96	5.9	3.5–10
**E28**	92	3.8	1.7–8.2
**E29**	92	2.3	0.75–5.7
**E30**	85	4.4	3.0–6.4
**E31**	89	4.4	2.1–9.6
**E32**	97	1.1	0.75–1.7
**E33**	92	0.95	0.070–3.5
**E34**	100	0.071	0.029–0.13
**E35**	100	0.18	0.10–0.33
**E36**	92	26	15–48
**E37**	95	0.19	0.13–0.26
**E38**	93	660	380–1100
**E39**	100	1.4	0.49–3.4

^a^Compounds were evaluated in *E*. *coli* reporter strain JLD271 [pJN105-cepR + pSC11-cepI]. Activity for 100 μM C_8_-AHL set to 100%. All assays n ≥ 3. Maximal activation error = ±10%. See Methods for full details of assay methods. ^b^CI = 95% confidence interval for EC_50_ value. ^c^Dose-response curve did not reach maximum plateau.

Regarding our choice of CepR for this Ec-reporter, *B*. *cenocepacia* is among the most common Bcc-isolate from CF patients^44^. Further, there is high sequence similarity among both the *cepI* promoter sequences and the CepR protein sequences in Bcc member species (~76% and ~95%, respectively; Fig. S3), and the *lux* box is 100% conserved, suggesting compounds identified for CepR in *B*. *cenocepacia* will be effective in targeting CepR from other Bcc member species.

### CepR agonism results in the Ec-reporter

We used the CepR Ec-reporter to perform a primary agonism screen at a single compound concentration (100 μM, or its solubility limit, indicated in Table S1). Thirty-one compounds displaying ≥70% agonistic activity in CepR (relative to C_8_-AHL) were identified and subjected to dose-response analyses using the Ec-reporter (Table I; see Table S2 for complete screening data and Fig. S3 for dose-response curves). We focus our analyses here on the most potent CepR agonists found in these dose-response studies.

Overall, the most potent class of CepR agonists were phenylpropionyl homoserine lactones (PPHLs). In fact, 11 PPHL compounds were more potent agonists than C_8_-AHL, CepR’s native ligand (**E34** > **E35** ≈ **E37** > **B7** > **E33** > **E32** ≈ **E39** > **E28** > **E30** ≈ **E31** > C_8_-AHL; Table 1). Strikingly, we found PPHL **E34**, with a *para*-methylsulfide substituent, was nearly 100-fold more potent than C_8_-AHL (Act % = 104, EC_50_ = 0.07 nM) in the Ec-reporter. Other notable CepR agonists that had sub-nanomolar potencies were **E35**, **E37**, **B7**, and **E33**. Overall, we found *para*-substituted PPHLs to be more potent than their *meta*-substituted analogs. For example, *para*-nitro PPHL **E37** was a more potent agonist than its *meta*-substituted analog **E38** (EC_50_ values of 0.19 nM vs. 660 nM, respectively), and this trend held true for **E35** vs. **E36**, and **B7** vs. **E31**, among others (Tables 1 and S1). Interestingly, these PPHL-type CepR agonists contained varied types of aryl substituents. Electron withdrawing groups, such as a nitro (**E37**) or trifluoromethyl (**E35**) group yielded potent agonists, as did electron donating groups, such as methoxy (**E28**) or methyl (**E26**) groups. However, it appears that some kind of substituent on the aryl group is necessary for potent CepR agonism, as the non-substituted PPHL (**B9**) had an EC_50_ ~1000 fold higher than those for substituted PPHLs. Replacement of the aryl group with a bulkier cyclohexyl group (to give **B11**) did lead to an increase in potency relative to **B9**, suggestive that sterics may be more important than electronics in this set of CepR agonists.

These assay data suggest that, out of the compounds screened, the PPHL scaffold is ideal for CepR activation, perhaps as it shares a similar tail length as the native ligand C_8_-AHL (~8 atoms). We observed that the incorporation of more or less atoms in the tail dramatically reduced compound potency. AHLs with 6, 10, and 12 carbons in the acyl chain only weakly to moderately activated CepR relative to C_8_-AHL (C_6_-AHL, Act % = 87, EC_50_ ≥ 20000 nM; C_10_-AHL, Act % = 87, EC_50_ = 109 nM; and C_12_-AHL: Act % = 98, EC_50_ = 310 nM; Table S2), and longer acyl chain lengths were inactive (i.e., C_14_-AHL and C_16_-AHL). In most cases, compounds with tail lengths shorter than C_8_-AHL, for example, C_4_-AHL and C_6_-AHL, were considerably less agonistic and were actually CepR antagonists instead (see below).

The agonism assay data provided additional insights into CepR’s preference for AHL-type agonists. In congruence with previous findings for other LuxR-type receptors^25^, the stereochemistry of the lactone ring was key to CepR activation, as compound **B1**, the D-isomer of C_8_-AHL, was inactive (Table S2). Sulfonamide AHL analogs also failed to activate CepR (i.e., **A11**, **A12**, and **A13**) and were found to antagonize CepR instead (see below; Table 2 and Fig. S3). Lastly, we found that a methylene at the 3-position of the acyl tail (as opposed to a 3-OH or 3-oxo) allowed for maximal CepR activation and potency (C_8_-AHL, Act % = 100, EC_50_ = 5.7 nM; 3’OH-C_8_-AHL, Act % = 84, EC_50_ = 210 nM; and 3’O-C_8_-AHL, Act % = 87, EC_50_ = 1200 nM; Table 1 and S2). None of the 20 lead CepR agonists were oxidized at carbon 3 of the tail.

**Table 2:** CepR antagonism data for selected compounds in the *E*. *coli* reporter^a^.

Compound	Maximal Inhibition (%)	IC_50_ (μM)	95% CI (μM)^b^
C_4_-AHL	58	20^c^	15–28
C_6_-AHL	83	7.5	5.7–9.8
3’O-C_8_-AHL	71	16^d^	—
**A11**	78	11 h	5.1–22
**A12**	68	0.38^c^	0.10–1.2^e^
**B12**	69	3.7^c^	2.7–5.2
**C6**	66	39^c^	26–63
**C1**	8	—	—
**C7**	89	5.9^d^	—
**C8**	79	2.7^c^	2.1–3.5
**C14**	46	1.4	0.30–11^e^
**E3**	77	3.3^c^	2.2–5.7
**E5**	81	2.7	1.2–10
**E7**	76	3.1^c^	2.2–4.4
**E10**	79	0.43^c^	0.20–1.1^e^
**E25**	70	3^c^	2.2–4.5
**S4**	72	32	18–63

^a^Compounds were evaluated in *E*. *coli* reporter strain JLD271 [pJN105-cepR + pSC11-cepI]. Activity for 100 μM C_8_-AHL set to 100%. All assays n ≥ 3. Maximal activation error = ±10%. See Methods for full details of assay methods. ^b^CI = 95% confidence interval for IC_50_ value. ^c^Dose–response antagonism curve showed inversion to agonism (i.e., non-monotonic behavior) at high compound concentrations. Concentrations at which CepR agonism was observed were excluded from IC_50_ calculations. See SI for additional details. ^d^Curve did not reach minimum plateau. ^e^|Hill slope| < 1.0, which affected 95% CI.

### CepR antagonism results in the Ec-reporter

To identify antagonists of CepR, we screened the AHL library (at 100 μM) in competition against the native ligand C_8_-AHL (at approximately twice its EC_50_ value, 0.01 μM) in the Ec-reporter. We found numerous strong CepR antagonists in the initial screen, with 23 compounds exhibiting ≥50% inhibition of CepR activity. The active compounds were subjected to dose-response analyses in the Ec-reporter to obtain IC_50_ values and assess their relative potencies (Table 2; see Table S3 for complete screening data and Fig. S3 for dose-response curves). One general trend that we noted at the outset was the majority of the antagonists had acyl tails of similar or shorter overall length than CepR’s native ligand, C_8_-AHL. From this set, the strongest antagonists could be divided into two classes: (i) “short alkyl tail” AHLs or analogs thereof, and (ii) phenylacetanoyl HLs (PHLs). The discovery that the latter compounds were CepR antagonists was perhaps not surprising, as we have found substituted PHLs to strongly antagonize the majority of the LuxR-type receptors studied in our laboratory to date^22,24,29,45,46^. Interestingly, the unsubstituted PHL, **C1**, was recently reported to be the natural AHL signal used by the prosthecate bacterium, *Prosthecomicrobium hirschii*^47^.

The most potent CepR antagonists in the short-alkyl tail class were C_4_-AHL, C_6_-AHL, **A11**, **A12**, **B12**, and **S4** (Tables 2 and S3). In this class, the most potent was the 8-atom tail sulfonamide analog of the native ligand C_8_-AHL, **A12** (Inh % = 68, IC_50_ = 0.38 μM). The 7-atom sulfonamide **A11** was also a CepR antagonist, although 100-fold less potent than **A12**. The lead CepR antagonists in the PHL class were **C8**, **C14**, **E3**, **E5**, and **E10**, with the most potent PHLs displaying low micromolar IC_50_ values (Table 2). Having a substituent on the PHL aryl ring was critical to activity, as the unsubstituted PHL (and natural signal) **C1** was inactive, paralleling the lack of agonistic activity for the unsubstituted PPHL **B9** (see above). However, for at least the PHLs tested here, neither the position nor the nature of the substituent on the PHL aryl group appeared to dramatically impact activity, ranging from electron-withdrawing (-Cl, **C6**) to electron-donating (-SCH_3_, **E7**), although PHLs with fluorine in any position were inactive as antagonists (e.g., **C2**, **C3**, and **C4**; Table S3). The 3-nitro PHL **C14** was among our most potent CepR antagonists; we previously have found **C14** to display a diverse range of activities in LuxR-type receptors, ranging from being a highly potent agonist in LuxR (*V*. *fischeri*)^25^ and ExpR2 (*P*. *carotovora*)^27^ to a potent antagonist in LasR (*P*. *aeruginosa*)^25^. Intriguingly, we found that analogs of **C14** with alternative head groups exhibited dramatic losses in activity (e.g., thiolactone **F10**, cyclopentyl **F18**, phenyl **F25**, ethyl ester **F40**, and nitro-phenyl **F55** analogs; Table S3). This trend suggests that the HL headgroup may be key to CepR antagonism by at least this PHL scaffold.

### Evaluation of lead CepR modulators in a member of the Bcc

We next sought to assess if the CepR modulators identified in the Ec-reporter maintained their activities in a member of the Bcc. For these studies, we generated a *B*. *multivorans cepI::lacZ* reporter organism (Bm-reporter) using standard allelic exchange methods (see Methods). Along with *B*. *cenocepacia*, *B*. *multivorans* is one of the most commonly isolated Bcc members^6,44^. We note that small molecules must contend with a different cellular environment in the native organism relative to *E*. *coli*^48^; however, as both CepR and the *cepI* promoter are highly conserved among Bcc species (Fig. S3), we reasoned that the majority of compounds identified in the Ec-reporter (containing *B*. *cenocepacia* versions of the *cepI* promoter and the *cepR* gene) would maintain their relative activity profiles in CepR in *B*. *multivorans*. Our Bm-reporter organism had a genomic replacement of its synthase gene *cepI* with *lacZ*, resulting in a transcriptional fusion of the *cepI* promoter region with *lacZ* that encodes β-galactosidase. Similar to the Ec-reporter, CepR binding to the *cepI* promoter then induces β-galactosidase production, allowing for the quantitation of CepR activity. Using the Bm-reporter organism, we observed robust activity for CepR in response to C_8_-AHL (EC_50_ = 0.7 nM, Table 3).

**Table 3:** CepR agonism data for selected compounds in the *B*. *multivorans* reporter^a^.

Compound	Maximal Activation (%)	EC_50_ (nM)	95% CI (nM)^b^
C_8_-AHL	100	0.70	0.030–2.3^c^
3’OH-C_8_-AHL	78	≥980^d^	—
3’O-C_8_-AHL	72	≥3500^d^	—
**B7**	82	0.24	0.11–0.64^c^
**CL**	80	940	—
**E26**	96	0.60	0.1–69^c^
**E30**	84	0.75	0.26–2.3^c^
**E31**	78	≥3400^d^	—
**E32**	110	0.080	0.029–0.24^c^
**E33**	94	0.15	0.028–0.56^c^
**E34**	110	0.94	0.45–2.1^c^
**E35**	98	0.022	0.0062–0.067^c^
**E36**	100	≥3900^d^	—
**E37**	91	0.0025	0.00020–0.0090^c^

^a^Compounds were evaluated in reporter strain *B*. *multivorans cepI::lacZ*. Activity for 100 μM C_8_-AHL set to 100%. All assays n ≥ 3. Error = ±10%. See Methods for full details of assay methods. ^b^CI = 95% confidence interval for EC_50_ value. ^c^|Hill slope| < 1.0, which affected 95% CI. ^d^Dose-response curve did not reach maximum plateau.

**Figure 2:**
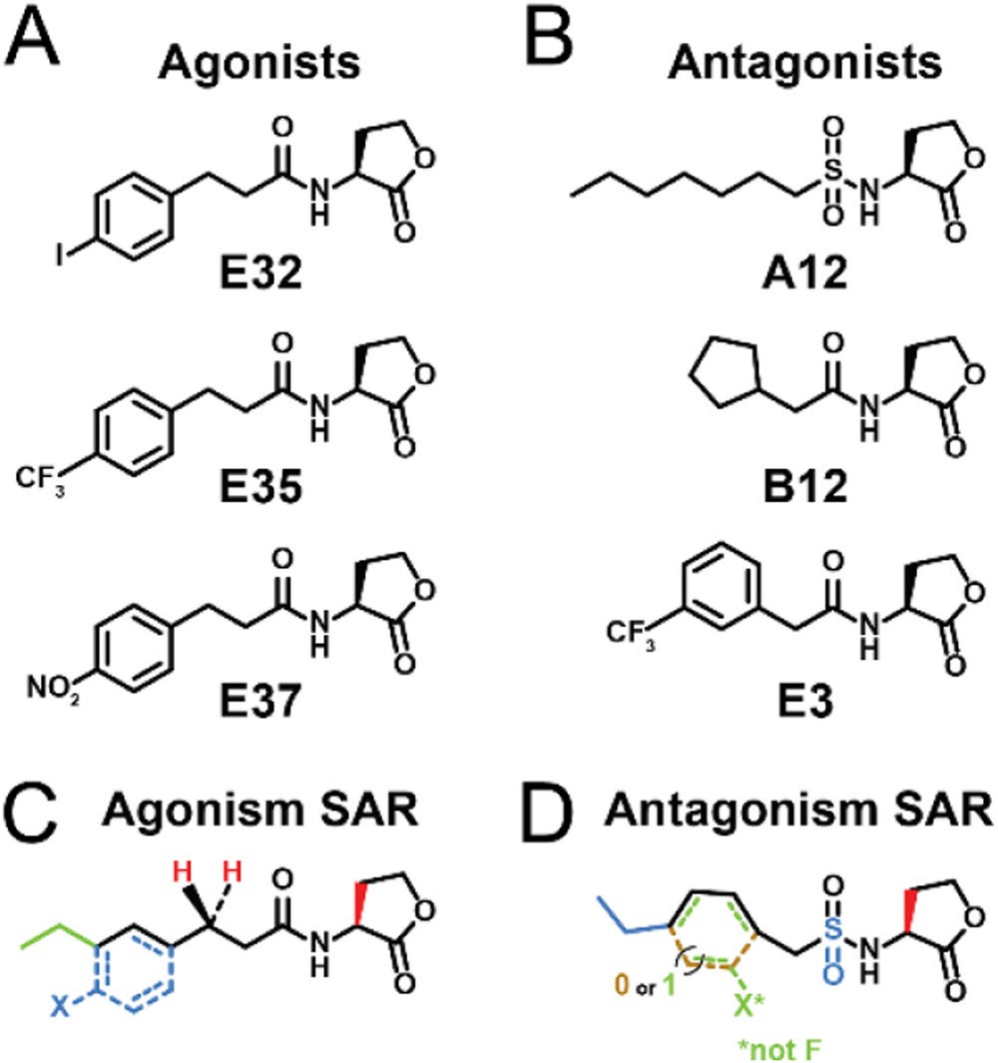
*Selected CepR modulators and SAR synopsis*. Lead CepR modulators and SAR for CepR (**A**) agonism and (**B**) antagonism. (**C**) Schematic indicating key features of the AHL scaffold that agonize CepR. Red indicate features found in all agonists. Blue indicates features of the strongest agonists. Green indicates native ligand. (**D**) Schematic indicating key features of the AHL scaffold that antagonize CepR. Red indicates features vital to antagonism. Blue indicates the sulfonamide scaffold. Green indicates the PHL scaffold. Brown indicates the cyclopentyl scaffold.

We screened the most potent AHLs identified in the Ec-reporter in this new Bm-reporter. Structures of the lead CepR agonists and antagonists identified are shown in Fig. 2A,B, and the SAR trends are summarized in Fig. 2C,D. The PPHLs retained the strongest CepR agonistic activities in the Bm-reporter overall (**E37** > **E35** > **E32** > **E33** > **B7** > **E30** ≈ **E26** ≈ C_8_-AHL). Most notably, we found *para*-nitro substituted PPHL **E37** especially potent in the Bm-reporter, with an EC_50_ ~250-fold lower than C_8_-AHL (Act % = 91, EC_50_ = 0.0025 nM). This potency improvement relative to the native ligand is notable and represents the largest increase, along with the lowest EC_50_ value overall (single digit picomolar), that we have measured in any LuxR-type receptor so far. When looking at overall potency trends between the Bm- and Ec-reporters, we note that the EC_50_ values were lower in the native organism; for example, the native ligand C_8_-AHL has an EC_50_ of 0.70 nM in the Bm-reporter, but has a ~10-fold higher EC_50_ (5.7 nM) in the Ec-reporter. PPHL **E34** remained a potent agonist; however, its activity was much closer to that of C_8_-AHL in the Bm-reporter. Of the agonists tested in both reporter assays, only the potencies of 3’OH-C_8_-AHL, 3’O-C_8_-AHL, **E31**, and **E36** were dramatically reduced in the Bm-reporter relative to the Ec-reporter.

Turning to the CepR antagonism data in the Bm-reporter, of the 10 most potent antagonists uncovered using the Ec-reporter, seven compounds remained in the top 10 in the Bm-reporter: **A12**, **B12**, **C8**, **C14**, **E3**, **E7**, and **E25** (Table 4). C4-AHL and the cyclopentyl HL **B12** were among the most potent antagonists in the Bm-reporter, supporting again that short-alkyl tails lead to CepR antagonism by AHLs. 3’-O-C_6_-AHL, the sole 3-oxo compound to have any activity in CepR, and the sulfonamide analog of C_8_-AHL, **A12**, both retained strong antagonistic activity in CepR. Likewise, PHLs **C14**, **C8**, **E3**, and **E25** remain potent antagonists for CepR in *B*. *multivorans*. Most of these PHLs were *meta*-substituted, yet with variable substituents. Notably, several PHL antagonists were able to bring reporter activity down to nearly zero in the Bm-reporter, with 3-bromo **C8**, 4-nitro **C13**, 3-CF_3_ **E3**, and 3-SMe **E7** among the most potent of this group. Overall, our screening data in the two reporter systems, each with different CepR receptors, suggest that ligands identified as active in one CepR receptor can be utilized to modulate other CepR homologs. These data also validate the use of the heterologous strain to identify CepR ligands that maintain their activity profiles in a native Bcc species.

**Table 4:** CepR antagonism data for selected compounds in the *B*. *multivorans* reporter^a^.

Compound	Maximal Inhibition (%)	IC_50_ (μM)	95% CI (μM)^b^
C_4_-AHL	76	0.25^c^	0.065–0.57^d^
C_6_-AHL	20	—	—
3’O-C_6_-AHL	85	0.80	0.20–62^d^
**A12**	82	1.7	0.93–3.0
**B12**	83	2.1	1.1–3.3
**C8**	94	2.6	1.2–4.5^d^
**C13**	96	3.6	1.5 to 95^d^
**C14**	54	0.62	0.30–1.5^d^
**E3**	100	1.9	1.1–3.9
**E5**	43	—	—
**E7**	97	4.0	2.2–13^d^
**E10**	21	—	—
**E13**	13	—	—
**E25**	83	3.3	2.0–8.4
**S4**	93	3.7	1.5–2^d^

^a^Compounds were evaluated in reporter strain *B*. *multivorans cepI::lacZ*. Activity for 100 μM C_8_-AHL set to 100%. All assays n ≥ 3. Error = ±10%. See Methods for full details of assay methods. ^b^CI = 95% confidence interval for IC_50_ value. ^c^Dose–response antagonism curve showed inversion to agonism (i.e., non-monotonic behavior) at high compound concentrations. Concentrations at which CepR agonism was observed were excluded from IC_50_ calculations. See SI for additional details. ^d^|Hill slope| < 1.0, which affected 95% CI.

### Swimming motility assays in *B*. *multivorans*

We were interested to assess the effects of our lead CepR modulators on a phenotype under the control of QS in *B*. *multivorans*. However, very little work has been reported to directly link CepR activity in *B*. *multivorans* to any QS-controlled phenotype. Motility has been shown to be controlled partially (*B*. *cenocepacia*)^49^ or completely (*B*. *cepacia* K56-2)^50,51^ by the CepI/CepR system in other Bcc members, and the *B*. *multivorans* organism has been shown to be motile^52^. Therefore, we chose to study the effects of compound treatment on both the swarming and swimming motility of *B*. *multivorans* (*B*. *multivorans* ATCC17616; see Methods).

**Figure 3:**
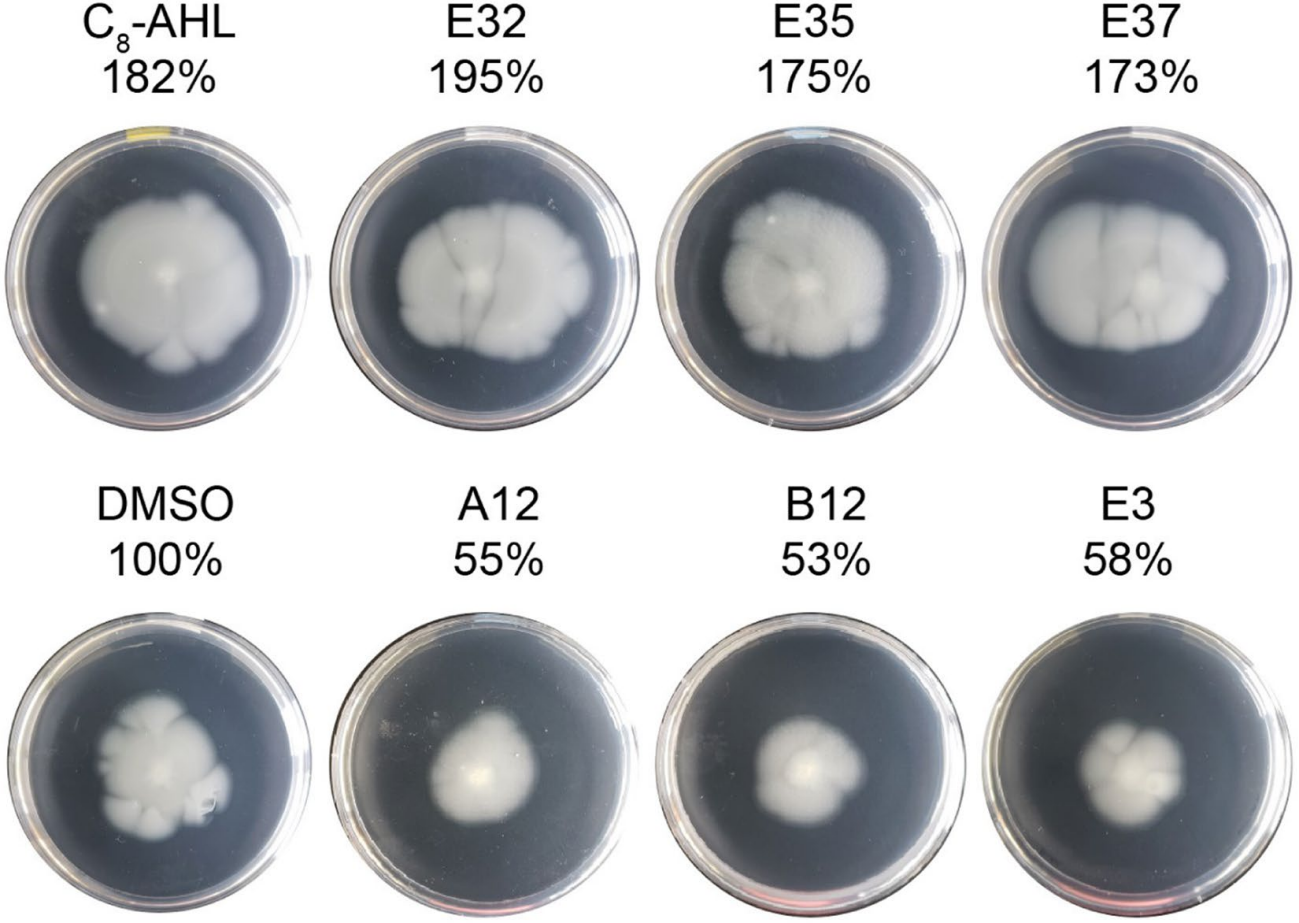
*B*. *multivorans motility assay data*. Swimming motility was measured by spotting *B*. *multivorans* plus compound indicated in the center of M9 + 0.1% casamino acids + 0.3% agar in a 10 cm diameter petri dish. Area of the colony after 72 h incubation was calculated using ImageJ, normalized to the *B*. *multivorans* wild-type + vehicle (DMSO), and listed above each plate shown.

Swarming motility (on 0.5% agar^52^) was not increased upon addition of C_8_-AHL, suggesting swarming is not under the direct control of CepI/CepR in *B*. *multivorans* (Fig. S4). In contrast, we found that under our swimming conditions (on 0.3% agar), the addition of native ligand C_8_-AHL increased swimming radius in both the parental and *cepI::lacZ* organisms (Figs 3 and S4), suggesting swimming motility is at least partially under the control of CepI/CepR in *B*. *multivorans*. We then tested the ability of our non-native compounds to modulate swimming motility in *B*. *multivorans*. Motility increased upon treatment by three of our most potent CepR agonists, **E32** (195%), **E35** (175%), and **E37** (173%), which was comparable to that by the native signal C_8_-AHL (182% relative to untreated sample, Fig. 3). In contrast, treatment with some of our strongest CepR antagonists, **A12**, **B12**, and **E3**, significantly decreased motility (55%, 53%, and 58%, respectively, relative to untreated sample). These results support that swimming motility in *B*. *multivorans* is at least partially under the control of the CepI/CepR QS system and that our compounds can be used to alter this behavior.

### *B. multivorans* infection assays in *C. elegans*

**Figure 4:**
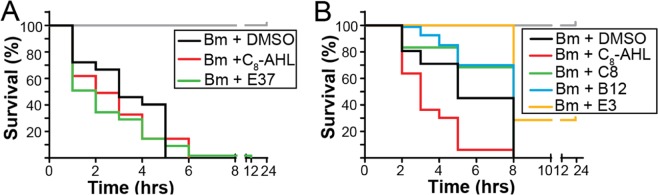
*C*. *elegans infection assay data*. Survival analysis of *B*. *multivorans* (Bm)-infected *C*. *elegans* N2 with treatment of the compound as indicated. For both plots, red lines indicate C_8_-AHL treatment, black lines indicate vehicle (DMSO) treatment, gray lines indicate compound treatment (lines overlap) but no bacteria. See Fig. S5 for full data and statistical analyses. (**A**) Survival plots of Bm-infected worms in the presence of strong agonist **E37** (green). (**B**) Survival plots of Bm-infected worms in the presence of antagonists **C8** (green), **B12** (blue) or **E3** (yellow).

We next assessed the effects of our lead CepR modulators on exacerbating or alleviating infections by a Bcc member using the nematode *C*. *elegans* as a model of pathogenicity. We infected N2 nematodes with *B*. *multivorans* ATCC17616 in the presence of compound, and assessed survival of worms over time (see Methods)^13,35,53^. None of the compounds tested caused measurable toxicity effects in uninfected worms under these conditions. Addition of CepR’s native ligand (C_8_-AHL) to Bm-infected worms significantly increased worm death relative to untreated worms (Fig. 4, p < 0.0001). Furthermore, addition of our strong CepR agonist **E37** promoted worm killing in *B*. *multivorans* infected worms in a similar manner (p < 0.0001). Weak CepR agonist **H2**, however, did not appear to affect worm survival under these conditions (Fig. S5, p = 0.69). Strikingly, many of the most potent CepR antagonists prolonged nematode survival; we show representative examples in Fig. 4 (**C8**, **B12**, and **E3**; for full set of survival assay data, see Fig. S5). Several previous studies, using genetic approaches, have demonstrated the importance of QS to pathogenicity in nematode infections in other members of the Bcc^13,54^. Our results here, using chemical CepR modulators, appear to replicate these effects in *B*. *multivorans* nematode infections. These data support chemical inhibition of CepR as a route to strongly attenuate virulence in a Bcc member.

## Discussion

Chemical strategies to modulate CepR-mediated QS in Bcc species would provide an approach to study the role of this signaling process in Bcc infections with both spatial and temporal control. Such chemical approaches are scarce, however, and no synthetic antagonists that both directly target CepR and also affect QS-controlled phenotypes have been reported to date. Here, we report CepR agonism and antagonism data for a library of synthetic AHL analogs obtained using both an *E*. *coli* reporter and a *B*. *multivorans* reporter. These small molecule screens identified a set of highly potent CepR activators and inhibitors. Furthermore, our lead compounds have the ability to alter QS-controlled phenotypes in wild-type *B*. *multivorans—*namely swimming motility and the survival of infected nematodes—in a CepR-dependent manner. Some general trends in the types of AHL structures that elicit CepR activation or inhibition are apparent (Fig. 2C,D). The most potent agonists contain aryl tail groups with various substitution patterns on the phenyl ring and a 2-carbon linker between the aryl ring and the amide bond. Among this class, the PPHL class stood out as a lead scaffold for CepR agonism. In turn, the most active CepR antagonists have an overall shorter acyl tail than the native ligand, C_8_-AHL, with various tail substituents, including aliphatic and phenylacetanoyl groups.

These SAR data for AHLs allowed us to consider potential mechanisms of action for their CepR agonism and antagonism. Agonism is perhaps the most straightforward to rationalize, as it is likely that these AHL derivatives act upon the same ligand-binding site in CepR as the native signal, C_8_-AHL. The only structural data of a C_8_-AHL-interacting LuxR-type receptor is for CepR2, consisting of an X-ray structure of its AHL-free, or “apo” ligand-binding domain^55^. Glycerol was found to be present in CepR2’s putative AHL-binding site and engaged with two key residues (Thr 130 and Asp 76) that have been found to be important for AHL binding in other LuxR-type receptors. In view of this similarity, we hypothesize that CepR binds C_8_-AHL and our non-native agonists using the same, conserved subset of amino acids as other LuxR-type receptors. We predict that CepR:AHL binding then leads to conformational changes within the protein to elicit homodimerization and downstream gene activation (similar to other LuxR-type receptors; see Fig. 1), and the 7–8 atom acyl chain length in our top CepR agonists is ideal for promoting such AHL-mediated CepR activation.

The mechanism of action for the CepR antagonists, however, remains ambiguous. The overall tail length of our most potent CepR antagonists was comparatively shorter than the native ligand. Plus, we observed that potent agonists could switch to antagonists by the subtraction of one carbon from the acyl chain (**B7** vs. **Control 7**, **E31** vs. **C8**, **E37** vs. **C13**, **E38** vs. **C14**, and **E36** vs. **E3** (Tables 1 and 2). These data suggest our antagonists may compete with the native ligand in binding CepR, yet upon binding cause different interactions that block activation. From here, whether the mechanism of CepR inhibition by these compounds is one of protein destabilization, as previously seen with LasR^56^ and QscR^57^ in *P*. *aeruginosa*, or protein stabilization in such a way as to inhibit its ability to bind DNA, as demonstrated with CviR from *C*. *violaceum*^58^, or a combination of pathways or a new pathway entirely, will require further experimentation.

To close, the compounds identified herein constitute new chemical tools to study QS in Bcc species in a variety of contexts. Given the strong conservation of CepR between Bcc members, we hypothesize our compounds will maintain their activity profiles across Bcc member species and we can begin to use them to assess the role of CepI/CepR QS on a broader scale. In addition, these compounds could prove useful when investigating polymicrobial infections involving Bcc members; for example, co-infections between *P*. *aeruginosa* and Bcc species in the CF lung. A next step moving forward will be to gauge the selectivity of the ligands identified herein for CepR over other LuxR-type receptors. When comparing the activities of our lead compounds in CepR vs. the three LuxR-type receptors in *P*. *aeruginosa* (i.e., LasR, RhlR, and QscR)^22,24,59^, our preliminary analyses show that agonist **E37**, and antagonists **A12** and **E3** are selective for CepR over these receptors. Such compounds could allow for the selective targeting of QS in one organism in a mixed culture over time and allow for insights into the potential for interspecies interactions via QS and their effects on host health outcomes. Ongoing studies are focused in this arena, along with further delineating the mechanisms by which our CepR ligands agonize and antagonize CepR, and will be reported in due course.

## Methods

### Bacterial strains and growth conditions

The bacterial strains and plasmids used in this study are summarized in Table S3. All biological media and reagents were obtained from commercial sources and used according to the manufacturer’s instructions. *E*. *coli* JLD271, *E*. *coli* OP50, and *B*. *multivorans* were grown in LB (Research Products International) at 37 °C with shaking (at 225 rpm). Bacterial growth was assessed by measuring the culture absorbance at 600 nm (OD_600_) using a Biotek Synergy 2 microplate reader operating Gen5 data analysis software. The *E*. *coli* reporter was grown in Luria Bertani (LB) medium containing 100-μg/mL ampicillin and 15-μg/mL gentamicin. The *B*. *multivorans* reporter was also grown in LB medium. Freezer stocks of bacterial strains were maintained at −80 °C in 1:1 LB:glycerol. *C*. *elegans* N2 was propagated under standard conditions, synchronized by hypochlorite bleaching, and cultured on nematode growth medium using *E*. *coli* OP50 as a food source, as described previously^53^.

### Chemicals, reagents, and compound handling

The non-native AHLs examined in this study were synthesized and purified as described previously^24,25,37,46,59,60^. The structures of all of the compounds are shown in Fig. S1. C_4_-AHL, C_6_-AHL, 3’O-C_6_-AHL, C_8_-AHL, 3’O-C_8_-AHL, 3’O-C_12_-AHL were purchased from Sigma-Aldrich. Chlorophenol red-β-D-galactopyranoside (CPRG), the substrate for the β-galactosidase assays, was purchased from Santa Cruz Biotechnology, Inc. All enzymes were purchased from New England Biolabs and used according to manufacturer’s instructions. Water (18 MΩ) was purified using a Millipore Feed System. Stock solutions of compounds were prepared in DMSO and stored at −20 °C in sealed glass vials. Final DMSO concentrations per well in biological assays did not exceed 1% (v/v). No compound had an effect on bacterial growth over the concentrations tested as gauged by monitoring OD_600_ over the time course of the assays.

### Ec-reporter plasmid construction

The pSC11-*cepI* reporter plasmid was constructed from pSC11-L^39^, using *B*. *cenocepacia* genomic DNA as a template (ATCC BAA-245D-5). Primers (9-SalI-cepI*-F and 10-BamHI-cepI’-R) containing restriction sites for SalI and BamHI were used to amplify the *cepI* genomic region using OneTaq, digested with SalI and BamHI, and cloned into pSC11 digested with the same enzymes using Quick Ligase. pSC11-*cepI* was sequence verified. The pJN105-*cepR* plasmid was constructed by modifying pJN105-L^40^. Primers (7-EcoRI-cepR-F and 8-XbaI-cepR-R) containing EcoRI and XbaI restriction sites were used to amplify *cepR* from *B*. *cenocepacia* genomic DNA (ATCC BAA-245D-5). The PCR product was digested with XbaI and EcoRI, then cloned into pJN105-L digested with the same enzymes using Quick Ligase. This plasmid was sequence verified (see Table 3; primers 2-pBADprom-F, 13-cepR-R2, and 14-cepR-F2).

### *B*. *multivorans cepI::lacZ* reporter organism construction

The *B*. *multivorans cepI::lacZ* strain was generated by replacing the native *cepI* with a promoterless *lacZ*. We first generated an intermediate plasmid, pEXG2 Δ*cepI* (Genscript), which had DNA fragments flanking *cepI* joined by two restriction enzyme recognition sequences, *xbaI* and *scaI*. These sites were used to introduce Klenow-blunted XbaI- and AseI-cut *lacZ* from pUC18-miniTn7T-Gm-lacZ^61^. The generated pEXG2 P*cepI::lacZ* plasmid was mated into *B*. *multivorans* ATCC17616 using the *E*. *coli* strain RHO3^62^, which requires diaminopimelic acid (DAP, added at 400 μg/mL final) for growth. *B*. *multivorans* exconjugants containing the chromosomally integrated pEXG2 P*cepI::lacZ* plasmid were isolated on LB agar plates using gentamicin at 300 μg/mL and no DAP for counterselection of *E*. *coli*. Strains in which the plasmid had excised were subsequently selected on no-salt LB agar plates containing 15% sucrose (no-salt LB agar plates containing 10 g bacto-tryptone, 5 g yeast extract, and 15 g agar/L of water). Mutants with the desired P*cepI::lacZ* allele were identified by the ability to turn blue on LB agar plates containing 40 μg/mL 5-bromo-4-chloro-3-indolyl-β-D-galactopyranoside (to measure CepR-induced β -galactosidase production) and 10 μM C_8_-HSL (to activate CepR), and verified by PCR amplification and sequencing of the PCR products.

### *E*. *coli* β-galactosidase reporter method

*E*. *coli* β-galactosidase reporter assays (derived from ref.^63^) and reagents used to obtain primary screening and dose-response data were performed as previously described^22,60,64^, with the following modifications: *E*. *coli* JLD271 (∆*sdiA*) was co-transformed iteratively with pSC11-*cepI* and pJN105-*cepR* via electroporation. A single colony was used to start overnight cultures in LB medium + ampicillin (100 μg/mL) + gentamicin (15 μg/mL). A 1:10 dilution of overnight culture was grown in fresh medium to OD_600_ = 0.25. A sterile solution of L-arabinose in water (27 mM final concentration) was added to induce protein expression, after which cells were plated in 96-well plates containing varying concentrations of compound (200 μL total volume).

Cells were grown for 4 h, OD_600_ was read, 50 μL aliquots from each well were permeabilized in 200 μL Z Buffer, and then 150 μL of this mixture used to measure the cleavage of 25 μL of 4 mg/mL CPRG in phosphate buffered saline (PBS) at 37 °C until positive control wells developed a deep red color (~30 min). For all agonism assays, synthetic compound (100 μM) was screened alongside and compared to the native ligand, C_8_-AHL (100 μM). For all primary and dose-response antagonism assays, the concentration of native ligand utilized was 10 nM (approximately equal to twice its EC_50_ value). IC_50_ and EC_50_ values, as well as 95% confidence intervals, were determined from sigmoidal curve fits using GraphPad Prism software (v.7.02, dose-response data found in Figs S3 and S4) using a variable slope parameter. At least 3 separate trials were performed using unique cultures on separate days.

### *Burkholderia multivorans cepI::lacZ* reporter assays

The *B*. *multivorans cepI::lacZ* strain was used to obtain dose-response data for CepR in response to synthetic AHLs. Overnight cultures of *B*. *multivorans cepI::lacZ* were grown in LB medium at 37 °C with shaking (225 rpm). Aliquots (200 μL) of a 1:10 dilution of this overnight culture were plated into 96-well plates containing varying concentrations of compound, and then grown for 4 h at 37 °C with shaking (225 rpm). β-galactosidase production was measured as described above for *E*. *coli*, with the following changes: permeabilized cells were incubated at 25 °C with substrate CPRG for ~10 min until positive control wells developed a deep red color. For agonism assays, synthetic compound was screened alongside and compared to the native ligand, C_8_-AHL (100 μM). For antagonism assays, the concentration of native ligand utilized was 5 nM (approximately equal to twice its EC_50_ value).

### Motility assay

The motility assay was based on a previously reported procedure^52^. *B*. *multivorans* was grown overnight with 50 μM compound at 37 °C and normalized to OD_600_ = 0.4, and then cells were washed with sterile water and resuspended with 50 μM fresh compound. Cells (5 μL) were spotted in the center of M9 + 0.1% Casamino Acids + 0.3% agar plates (100 mm diameter). Cells were grown for 72 h at 37 °C. Images were analyzed using ImageJ to calculate the area of the colony and normalized to the area of *B*. *multivorans* wild-type + vehicle (DMSO).

### ***C***. ***elegans*survival assay**

The *C*. *elegans* survival assay was based on previously reported procedures^35,65^. To synchronize worms, eggs were harvested by hypochlorite bleaching (5 min in 1 mL 5% bleach, 0.25 mL 5 M NaOH, 3.75 mL H_2_O), and grown on Nematode Growth Media (NGM) + *E*. *coli* OP50 at 21 °C. *B*. *multivorans* was grown overnight with the compound of interest in LB medium (1 μM for agonists, 50 μM for antagonists), and then the overnight culture was centrifuged, resuspended in the assay medium, standardized to OD_600_ = 0.8, and 25 μL of this culture was used per condition. CFUs of these standardized cultures were used to confirm that bacteria numbers were relatively the same. For the survival assay, synchronized worms (L4 stage) were suspended in liquid NGM media containing bacteria and compound in 96-well plates (100 μL total volume). In all cases, DMSO volume did not exceed 1% of total volume. Plates were incubated at 25 °C, after which they were analyzed by a dissecting microscope. The fraction of dead worms was determined by scoring the number of dead worms and the total number of worms in each well. At least 30 nematodes were used for each well, and each assay was performed at least in duplicate on a single day (n ≥ 60). Survival assays were then repeated at least once on different days to verify patterns. Compound effect on worm survival rarely varied between replicates, but the overall worm susceptibility varied among assays. Kaplan-Meier survival curves were calculated using GraphPad Prism (v. 7.0) software.

## Supplementary information

Supplementary Information

## Data Availability

The datasets generated and analyzed during the current study are available from the corresponding author upon reasonable request.
