# Supplementary material for: Potent modulation of the CepR quorum sensing receptor and virulence in a Burkholderia cepacia complex member using non-native lactone ligands
Source: Sci Rep. 2019 Sep 17;9:13449. doi: 10.1038/s41598-019-49693-x (PMC6748986; doi:10.1038/s41598-019-49693-x)
Supplement: Supplementary file 1 — Supplementary Information [file 41598_2019_49693_MOESM1_ESM.pdf]

SUPPLEMENTAL INFORMATION

Potent modulation of the CepR quorum sensing receptor and virulence in a *Burkholderia cepacia* complex member using non-native lactone ligands

Betty L. Slinger,[1] Jacqueline J. Deay,[2] Josephine R. Chandler,[2] and Helen E. Blackwell[1,*]

[1]*Department of Chemistry, University of Wisconsin–Madison, 1101 University Ave., Madison, WI 53706 USA;* [2]*Department of Molecular Biosciences, University of Kansas, 1200 Sunnyside Ave., Lawrence, KS 66045 USA*

Correspondence: blackwell@chem.wisc.edu

Contents.

- ***Figure S1.*** Structures of AHLs and other QS modulators examined in this study.
- ***Table S1.*** Alternative compound names.
- ***Table S2.*** CepR agonism data for all compounds in the *E. coli* reporter.
- ***Table S3***. CepR antagonism data for all compounds in the *E. coli* reporter.
- ***Table S4.*** CepR agonism data for selected compounds in the *B. multivorans* reporter.
- ***Table S5.*** CepR antagonism data for selected compounds in the *B. multivorans* reporter.
- ***Figure S2.*** Dose-response data for lead compounds examined in this study.
- ***Figure S3.*** *cepI* promoter and CepR conservation analysis.
- ***Figure S4.*** Motility assays with *B. multivorans*.
- ***Figure S5.*** *C. elegans* survival assays with *B. multivorans* and lead compounds in this study.
- ***Figure S6.*** *C. elegans* survival assays with *B. multivorans* and *B. multivorans cepI::lacZ* +/- C8-AHL.
- ***Table S6.*** Strains and plasmids used in this study.
- **References**

Generic Library Structure

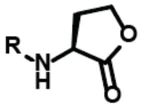

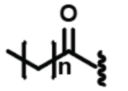

C_4-AHL: n = 2
C_6-AHL: n = 4
C_7-AHL: n = 5
C_8-AHL: n = 6
C_{10}-AHL: n = 8
C_{10}-CPA: n = 10
C_{12}-AHL: n = 10
C_{14}-AHL: n = 12
C_{16}-AHL: n = 14

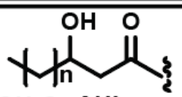

3'OH-C_6-AHL: n = 2
3'OH-C_8-AHL: n = 4
3'OH-C_{12}-AHL: n = 8
3'OH-C_{14}-AHL: n = 10
3'OH-C_{16}-AHL: n = 12

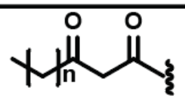

3'O-C_6-TL: n = 2
3'O-C_8-AHL: n = 4
3'O-C_8-AHL[i]: n = 4
3'O-C_8-TL: n = 4
3'O-C_{10}-AHL: n = 6
3'O-C_{12}-AHL: n = 8
3'O-C_{12}-analine: n = 8
3'O-C_{16}-AHL: n = 12

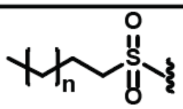

A11: n = 3; C_6
A12: n = 4; C_7
A13: n = 5; C_8

PHLs

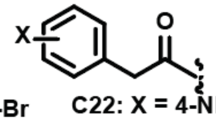

B4[i]: X = 4-Br
C1: X = H
C2: X = 4-F
C3: X = 3-F
C4: X = 2-F
C5: X = 4-Cl
C6: X = 3-Cl
C7: X = 2-Cl
C8: X = 3-Br
C9: X = 2-Br
C10: X = 4-I
C11: X = 3-I
C12: X = 2-I
C13: X = $4\text{-}NO_2$
C14: X = $3\text{-}NO_2$
C15: X = $2\text{-}NO_2$
C16: X = F_5
C17: X = $4\text{-}N_3$
C18: X = 4-Ph
C19: X = $4\text{-}CH_3$
C20: X = $4\text{-}CF_3$
C21: X = NH_2
C22: X = 4-NHBoc
C23: X = 4-OH
C24: X = $O\text{-}CH_3$
Control 7: X = 4-Br
C25: X = $O\text{-}CH_2CH_3$
E1: X = $3\text{-}CH_3$
E2: X = $3\text{-}OCH_3$
E3: X = $3\text{-}CF_3$
E4: X = 4-CN
E5: X = 3-CN
E6: X = $4\text{-}SCH_3$
E7: X = $3\text{-}SCH_3$
E8: X = $4\text{-}SCF_3$
E9: X = $3,5\text{-}CF_3$
E10: X = $3\text{-}CF_3$, 4-F
E11: X = 3,4-Cl
F10: TL, X = 4-Br
F11: TL, X = $3\text{-}NO_2$
F12: TL, X = $4\text{-}O\text{-}CH_3$
F13: TL, X = 4-Ph
F16: CPA, X = H
F18: CPA, X = $3\text{-}NO_2$
F20: CPA, X = 4-Ph
F25[ii]: X = $3\text{-}NO_2$
F39[iii]: X = 4-Br
F40[iii]: X = $3\text{-}NO_2$
F55[iv]: X = $3\text{-}NO_2$

POHLs

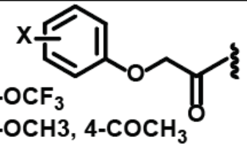

D15: X = $4\text{-}OCF_3$
D16: X = 2-OCH3, $4\text{-}COCH_3$
E14: X = H
E15: X = $4\text{-}CH_3$
E16: X = $3\text{-}CH_3$
E17: X = $4\text{-}OCH_3$
E18: X = $3\text{-}OCH_3$
E19: X = 4-F
E20: X = 4-Cl
E21: X = 4-Br
E22: X = 4-I
E23: X = $4\text{-}CF_3$
E24: X = $4\text{-}NO_3$
E25: X = $3\text{-}NO_2$

PHHLs

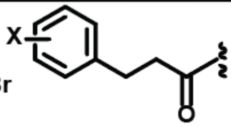

B7: X = 4-Br
B9: X = H
E26: X = $4\text{-}CH_3$
E27: X = $E\text{-}CH_3$
E28: X = $4\text{-}OCH_3$
E29: X = 4-F
E30: X = 4-Cl
E31: X = 3-Br
E32: X = 4-I
E33: X = 3-I
E34: X = $4\text{-}SCH_3$
E35: X = $4\text{-}CF_3$
E36: X = $3\text{-}CF_3$
E37: X = $4\text{-}NO_2$
E38: X = $3\text{-}NO_2$
E39: X = $3\text{-}OCH_2O\text{-}4$
F45[iii]: X = 3-I
F47[iii]: X = $4\text{-}OCH_3$

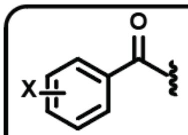

B6: X = 4-Br
B8: X = H
D10: X= 4-1,2,3 thiadiazole
D13: X = $4\text{-}C_8H_{17}$
D20: X = NNPh
Q1: X = 2-Br
Q2: X = 3-Br
Q3: X = 4-F
Q4: X = 4-Cl
Q5: X = 4-I
Q6: X = $4\text{-}OCH_3$
Q7: X = $4\text{-}NO_2$
Q8: X = $4\text{-}CH_3$
Q9: X = 4-oxtyloxy
Q11: X = $3\text{-}OCH_2O\text{-}4$
Q12: X = 4-Ph
Q13: X = 4-Boc
R1: X = 4-ethyloxy
R2: X = 4-propyloxy
R3: X = 4-isopropyloxy
R4: X = 4-butyloxy
R5: X = 4-pentyloxy

3'O-C_{12}-AHL with alt. head groups

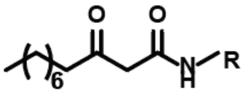

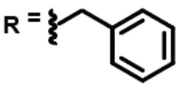

Control 13: n=0, X = $3\text{-}OCH_3$
Control 14: n=0, X = 2-OH
F26: n = 1, X = 2-F
F31: n = 1, X = $4\text{-}O\text{-}CH_3$
F50: n = 2

Control 15

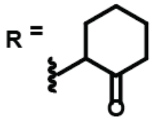

Branched AHLs

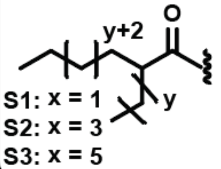

S1: x = 1
S2: x = 3
S3: x = 5

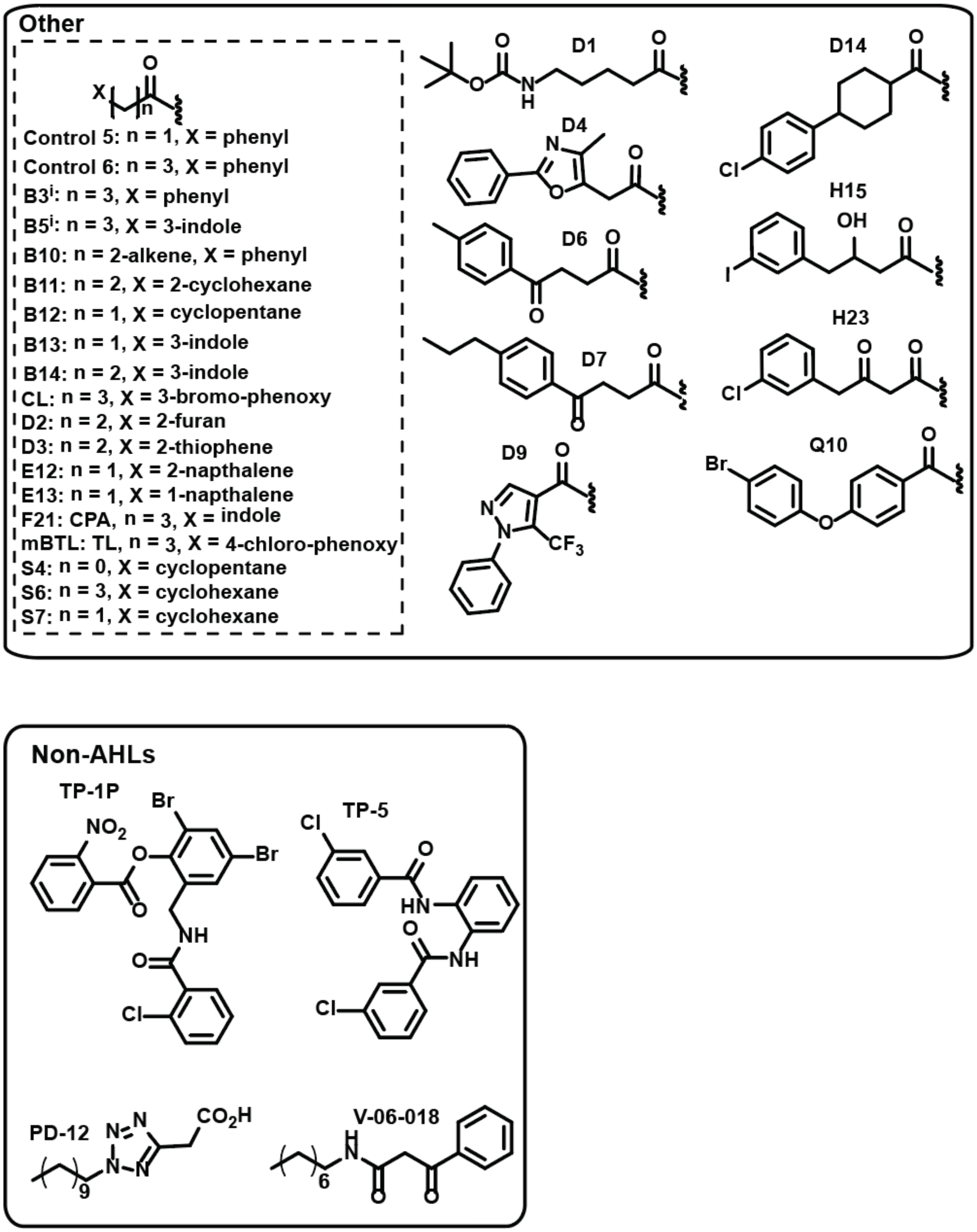

***Figure S1*.** Structures of AHLs and other QS modulators examined in this study. Abbreviations: [i]=inverted lactone head group stereochemistry; [ii]=phenyl head group; [iii]=ethyl ester head group; [iv]=3-nitro phenyl head group; TL=thiolactone head group; CPA=cyclopentyl amine head group.

Notes on compounds in Figure S1.

The compound numbering in Figure S1 matches that used in our previous reports. Compounds labeled as "control" compounds (e.g., Control 5) were from a collection of compounds we previously investigated for their activity against LasR in *P. aeruginosa*.[1] The A library (**A1**–**A7** and **A11**–**A13**) was composed of straight-chain AHLs of varied acyl chain length (4–16 carbons) and 3'-position oxidation state (methylene and oxo).[2] The B library (**B1** and **B3**–**B14**) contained cycloalkyl- and aromatic-containing AHLs and analogs of the AHLs with one-carbon insertions or deletions in the acyl chain or opposite lactone stereochemistry.[2] The C library (**C1**–**C25**) was composed of phenylacetanoyl homoserine lactones (PHLs) with substituents on the aryl ring of varying electronics and size, and in varying positions.[2] The D library (**D1**–**D4**, **D6**–**D7**, **D9**, **D10**, **D13**–**D16**, and **D20**) was composed of a wide variety of AHLs intended to broadly examine the effect of acyl chain structure on AHL activity.[3] The E library (**E1**–**E39**) contained additional PHLs and phenoxyacetanoyl homoserine lactones (POHLs) and phenylpropionyl HLs (PPHLs) with aryl ring substituents complementary to those in the C library.[4] The F library (**F2**, **F3**, **F10**–**F13**, **F16**, **F18**, **F20**, **F21**, **F25**, **F26**, **F31**, **F39**, **F40**, **F45**, **F47**, **F50**, and **F55**) contained a variety of AHL analogs, including several with non-native headgroups in place of the lactone.[5] The H library (**H2**, **H4**–**H6**, **H15**, **H23**, **H25**, and **H26**) contained AHLs with long acyl chains, aryl side chains, and/or 3'-hydroxyl groups.[6] The Q library (**Q1**–**Q13**) was composed of benzoyl homoserine lactones with varied substituents on the aryl ring.[7] The R library (**R1**–**R5**) contained *para*-alkoxy substituted benzoyl homoserine lactones of varying chain lengths.[7] The S library (**S1**–**S4**, **S6**, and **S7**) contained AHLs with either branched alkyl chains or cycloalkyl rings.[7] We selected 169 compounds from these libraries for the current study.

Table S1. Alternative compound names.

Referred to in text as:	Alternative name 1	Alternative name 2	Ref.
C_4-AHL	**A1**	butanoyl L-homoserine lactone (BHL)	
C_6-AHL	**A2**	hexanoyl L-homoserine lactone (HHL)	
C_7-AHL	Control 4		
C_8-AHL	**A3**	octanoyl L-homoserine lactone (OHL)	
C_{10}-AHL	**A4**	decanoyl L-homoserine lactone (DHL)	
C_{10}-CPA	Control 17		
C_{12}-AHL	**A5**	Dodecanoyl L-homoserine lactone (dDHL)	
C_{14}-AHL	**H26**	tetradecanoyl L-homoserine lactone (tDHL)	
C_{16}-AHL	**A6**	hexadecanoyl L-homoserine lactone (hDHL)	
3'OH-C_6-AHL	Control 3	OHHL	
3'OH-C_8-AHL	**H2**		
3'OH-C_{12}-AHL	**H4**		
3'OH-C_{14}-AHL	**H5**		
3'OH-C_{16}-AHL	**H6**		
3'O-C_6-TL	**F3**		
3'O-C_8-AHL	Control 2		
D-3'O-C_8-AHL	**B1**		
3'O-C_8-TL	**F2**		
3'O-C_{10}-AHL	**A7**		
3'O-C_{12}-AHL	Control 1		
3'O-C_{12}-analine	Control 12		[5]
3'O-C_{16}-AHL	**H25**		
**B7**	Control 8		[8]
Chlorolactone (CL)	Control 10		[9]
**E35**	Control 9		[8]
meta-bromo-thiolactone (mBTL)	Control 16		[10]
PD-12	Control 22		[11]
TP-1P	Control 19		[12]
TP-5	Control 20		[13]
V-06-018	Control 18		[10]

Table S2. CepR agonism data for all compounds tested in the *E. coli* reporter.

**Compound**	**Screening Conc. [μM]**	**Act%**	**EC_{50} [nM]**	**95% CI [nM]**
C_4-AHL	100	23		
C_6-AHL	100	87	≥20000	
3'O-C_6-TL	100	1.6		
3'OH-C_6-AHL	100	0.0		
C_7-AHL	100	106	≥930	
C_8-AHL	100	100	5.7	4.6-6.9
3'OH-C_8-AHL	100	84	210	160-290
3'O-C_8-AHL	100	86	1200	720-2600
D-3'O-C_8-AHL	100	85	≥27000	
3'O-C_8-TL	100	61		
C_{10}-AHL	100	87	110	83-150
C_{10}-CPA	100	48		
3'O-C_{10}-AHL	100	110	2300	1500-4200
C_{12}-AHL	100	98	310	180-530
3'OH-C_{12}-AHL	100	52		
3'O-C_{12}-AHL	100	98	≥4400	
3'O-C_{12}-analine	100	52		
C_{14}-AHL	100	49		
3'OH-C_{14}-AHL	100	33		
C_{16}-AHL	25	35		
3'OH-C_{16}-AHL	100	8.0		
**A11**	100	-5.0		
**A12**	100	46		
**A13**	100	108	150	100-240
**B3**	100	-0.4		
**B4**	100	-0.5		
**B5**	100	7.2		
**B6**	100	56		
**B7**	100	97	0.29	0.14-0.59
**B8**	100	10		
**B9**	100	88	1000	590-2100
**B10**	100	60		
**B11**	100	87	130	85-200
**B12**	100	39		
**B13**	100	-1.1		
**B14**	100	96	≥4300	
CL	100	110	12	7.5-18
Control 5	100	9.8		
Control 6	100	80	≥39000	
Control 7	100	0.2		

Control 13	100	2.0		
Control 14	100	30		
Control 15	100	5.2		
**C1**	100	0.0		
**C2**	100	0.1		
**C3**	100	0.2		
**C4**	100	0.1		
**C5**	100	0.1		
**C6**	100	3.2		
**C7**	100	0.3		
**C8**	100	12		
**C9**	100	0.3		
**C10**	100	29		
**C11**	100	43		
**C12**	100	-0.4		
**C13**	100	1.1		
**C14**	100	16		
**C15**	100	1.4		
**C16**	100	0.1		
**C17**	100	1.0		
**C18**	100	1.4		
**C19**	100	-1.6		
**C20**	100	0.3		
**C21**	100	21		
**C22**	100	1.1		
**C23**	100	-0.5		
**C24**	100	0.1		
**C25**	100	-1.6		
**D1**	100	16		
**D2**	100	70		
**D3**	100	87	≥33000	
**D4**	100	40		
**D6**	100	61		
**D7**	100	18		
**D9**	100	14		
**D10**	100	43		
**D13**	100	4.2		
**D14**	40	54		
**D15**	100	46		
**D16**	100	3.5		
**D20**	77	37		
**E1**	100	16		
**E2**	100	28		

**E3**	100	6.6		
**E4**	100	0.2		
**E5**	100	4.9		
**E6**	100	3.7		
**E7**	100	5.9		
**E8**	100	14		
**E9**	100	2.2		
**E10**	100	13		
**E11**	100	1.9		
**E12**	100	11		
**E13**	100	2.5		
**E14**	100	57		
**E15**	100	65		
**E16**	100	11		
**E17**	100	50		
**E18**	100	30		
**E19**	100	42		
**E20**	100	100	210	
**E21**	100	89	≥17000	
**E22**	100	92	≥5500	
**E23**	100	89	≥16000	
**E24**	100	75	≥15000	
**E25**	100	25		
**E26**	100	96	5.9	3.5-10
**E27**	100	75	850	490-1800
**E28**	100	92	3.8	1.7-8.2
**E29**	100	92	2.3	0.75-5.7
**E30**	100	85	4.4	3.0-6.4
**E31**	100	89	4.4	2.1-9.6
**E32**	100	97	1.1	0.75-1.7
**E33**	100	92	0.95	0.070-3.5
**E34**	100	104	0.071	0.029-0.13
**E35**	100	100	0.18	0.097-0.33
**E36**	100	92	26	15-48
**E37**	100	95	0.19	0.13-0.26
**E38**	100	93	660	380-1100
**E39**	100	103	1.4	0.50-3.4
**F10**	100	0.5		
**F11**	100	45.0		
**F12**	100	2.4		
**F13**	100	35		
**F16**	100	7.4		
**F18**	100	1.0		

**F20**	100	0.5		
**F21**	100	-4.0		
**F25**	100	3.0		
**F26**	100	-1.2		
**F31**	100	7.6		
**F39**	100	10		
**F40**	100	16		
**F45**	100	16		
**F47**	100	24		
**F50**	50	12		
**F55**	100	25		
**H15**	100	2.6		
**H23**	100	53		
**H25**	100	8.3		
mBTL	100	0.4		
PD-12	100	-7.6		
**Q1**	100	5.8		
**Q2**	100	4.9		
**Q3**	100	11		
**Q4**	100	49		
**Q5**	100	78	≥3400	
**Q6**	100	25		
**Q7**	100	10		
**Q8**	100	19		
**Q9**	100	62		
**Q10**	100	64		
**Q11**	100	6.9		
**Q12**	100	111	≥2800	
**Q13**	100	6.8		
**R1**	100	99	180	
**R2**	100	98	38	23-65
**R3**	100	92	94	49-200
**R4**	25	84	86	49-170
**R5**	100	95	≥910	
**S1**	100	48		
**S2**	100	35		
**S3**	100	86	5100	3400-7100
**S4**	100	0.2		
**S6**	100	90	≥38000	
**S7**	100	7.6		
TP-1P	100	54		
TP-5	100	0.4		
V-06-018	100	2.4		

Table S3. CepR antagonism data for all compounds tested in the *E. coli* reporter.

**Compound**	**Screening Conc. [μM]**	**Inh% (100-Act%)**	**IC_{50} [μM]**	**95% CI [μM]**
C_4-AHL	100	58	20	15-28
C_6-AHL	100	83	7.5	5.7-9.8
3'OH-C_6-AHL	100	71	≥16	
3'O-C_6-TL	100	4.0		
C_7-AHL	100	-10		
C_8-AHL	100	-30		
3'OH-C_8-AHL	100	-20		
3'O-C_8-AHL	100	3.0		
D-3'O-C_8-AHL	100	-40		
3'O-C_8-TL	100	-20		
C_{10}-AHL	100	-30		
C_{10}-CPA	100	4.0		
3'O-C_{10}-AHL	100	-60		
C_{12}-AHL	100	-30		
3'OH-C_{12}-AHL	100	0.0		
3'O-C_{12}-AHL	100	-40		
3'O-C_{12}-analine	100	16		
C_{14}-AHL	100	-10		
3'OH-C_{14}-AHL	100	-40		
C_{16}-AHL	25	-10		
3'OH-C_{16}-AHL	100	-50		
3'O-C_{16}-AHL	100	-50		
**A11**	100	78	≥11	
**A12**	100	68	0.38	0.10-1.2
**A13**	100	17		
**B3**	100	-30		
**B4**	100	0.0		
**B5**	100	-30		
**B6**	100	18		
**B7**	100	-50		
**B8**	100	1.0		
**B9**	100	-40		
**B10**	100	11		
**B11**	100	-20		
**B12**	100	69	3.7	2.7-5.2
**B13**	100	22		
**B14**	100	-40		
CL	100	-20		
Control 5	100	11		
Control 6	100	3.0		

Control 7	100	89	≥20000	
Control 13	100	13		
Control 14	100	30		
Control 15	100	29		
**C1**	100	8.0		
**C2**	100	4.0		
**C3**	100	20		
**C4**	100	3.0		
**C5**	100	25		
**C6**	100	66	39	263
**C7**	100	89	≥5.9	
**C8**	100	79	2.7	2.1-3.5
**C9**	100	52	40	22-79
**C10**	100	-10		
**C11**	100	27		
**C12**	100	72	22	15-30
**C13**	100	79	6.7	4.8-9.7
**C14**	100	46	1.4	0.30-11
**C15**	100	79	≥10	
**C16**	100	-30		
**C17**	100	26		
**C18**	100	-20		
**C19**	100	-20		
**C20**	100	-52		
**C21**	100	-20		
**C22**	100	-70		
**C23**	100	-10		
**C24**	100	-60		
**C25**	100	0.0		
**D1**	100	11		
**D2**	100	0.0		
**D3**	100	-40		
**D4**	100	22		
**D6**	100	-50		
**D7**	100	15		
**D9**	100	0.0		
**D10**	100	0.0		
**D13**	100	38		
**D14**	40	-20		
**D15**	100	-20		
**D16**	100	0.0		
**D20**	77	8.0		
**E1**	100	11		
**E2**	100	-10		

**E3**	100	77	3.3	2.2-5.7
**E4**	100	29		
**E5**	100	81	2.7	1.2-10
**E6**	100	-10		
**E7**	100	76	3.1	2.2-4.4
**E8**	100	0.0		
**E9**	100	13		
**E10**	100	79	0.43	0.20-1.1
**E11**	100	59	≥18	
**E12**	100	-10		
**E13**	100	50	4.9	1.2-23
**E14**	100	-30		
**E15**	100	-30		
**E16**	100	21		
**E17**	100	0.0		
**E18**	100	0.0		
**E19**	100	22		
**E20**	100	-20		
**E21**	100	-10		
**E22**	100	-30		
**E23**	100	-10		
**E24**	100	-10		
**E25**	100	70	3	2.2-4.5
**E26**	100	-10		
**E27**	100	-10		
**E28**	100	-30		
**E29**	100	-10		
**E30**	100	-10		
**E31**	100	-20		
**E32**	100	-20		
**E33**	100	-20		
**E34**	100	-20		
**E35**	100	-50		
**E36**	100	-10		
**E37**	100	-30		
**E38**	100	-10		
**e39**	100	-40		
**F10**	100	6.0		
**F11**	100	25		
**F12**	100	-20		
**F13**	100	-20		
**F16**	100	-30		
**F18**	100	-20		
**F20**	100	-10		
**F21**	100	25		

**F25**	100	12		
**F26**	100	20		
**F31**	100	21		
**F39**	100	17		
**F40**	100	16		
**F45**	100	19		
**F47**	100	12		
**F50**	50	1.0		
**F55**	100	13		
**H15**	100	18		
**H23**	100	-20		
mBTL	100	-60		
PD-12	100	-40		
**Q1**	100	-30		
**Q2**	100	-30		
**Q3**	100	17		
**Q4**	100	-20		
**Q5**	100	0.0		
**Q6**	100	-20		
**Q7**	100	28		
**Q8**	100	-20		
**Q9**	100	-20		
**Q10**	100	-40		
**Q11**	100	5.0		
**Q12**	100	-70		
**Q13**	100	11		
**R1**	100	-20		
**R2**	100	-10		
**R3**	100	-10		
**R4**	25	-30		
**R5**	100	-10		
**S1**	100	-50		
**S2**	100	8.0		
**S3**	100	-50		
**S4**	100	72	32	18-63
**S6**	100	-10		
**S7**	100	20		
TP-1P	100	0.0		
TP-5	100	24		
V-06-018	100	-20		

Table S4. CepR agonism data for all compounds tested in the *B. multivorans* reporter.

**Compound**	**Screening Conc. [µM]**	**Act%**	**EC_{50} [nM]**	**95% CI [nM]**
C_8-AHL	100	100	0.70	0.030-2.3
3'OH-C_8-AHL	100	78	≥980	
3'O-C_8-AHL	100	72	≥3500	
3'O-C_{12}-AHL	100	80	≥20000	
**B7**	100	82	0.24	0.11-0.64
**B11**	100	51	n/a	
CL	100	80	940	
**C11**	100	2.3	n/a	
**E3**	100	3.1	n/a	
**E10**	100	11	n/a	
**E26**	100	96	0.77	
**E28**	100	90	52.0	20-150
**E29**	100	94	≥800000	
**E30**	100	84	0.75	0.26-2.3
**E31**	100	78	≥3400	
**E32**	100	110	0.080	0.029-0.24
**E33**	100	94	0.15	0.028-0.56
**E34**	100	110	0.94	0.45-2.1
**E35**	100	98	0.022	0.0062-0.067
**E36**	100	103	≥3900	
**E37**	100	91	0.0025	0.00020-0.0090
**E39**	100	84	9.1	1.4-3400
**R2**	100	92	≥160	
**R3**	100	40		
**R4**	25	58		

Table S5. CepR antagonism data for all compounds tested in the *B. multivorans* reporter.

**Compound**	**Screening Conc. [μM]**	**Inh% (100-Act%)**	**IC_{50} [μM]**	**95% CI [μM]**
C_4-AHL	100	76	0.25	0.065-0.57
C_6-AHL	100	20		
3'O-C_6-AHL	100	85	0.80	0.20-62
**A11**	100	84	≥18	
**A12**	100	82	1.7	0.93-3
**B12**	100	83	2.1	1.1-3.3
Control 7	100	85	≥11	
Control 11	100	44		
Control 14	100	25		
Control 20	100	15		
**C6**	100	98	≥7.0	
**C7**	100	95	≥13	
**C8**	100	94	2.6	1.2-4.5
**C9**	100	99	≥11	
**C11**	100	80	1.6	0.85-3.4
**C12**	100	104	≥27	
**C13**	100	96	3.6	1.5-32
**C14**	100	54	0.62	0.30-1.5
**C15**	100	95	≥11	
**E3**	100	100	1.9	1.1-3.9
**E4**	100	97	≥4.5	
**E5**	100	43		
**E7**	100	97	4.0	2.2-13
**E10**	100	21		
**E11**	100	94	≥12	
**E13**	100	13		
**E25**	100	83	≥3.3	
**S4**	100	93	3.7	1.5-21

Figure S2. Dose-response data for compounds examined in this study.

<u>*Ec-reporter CepR agonist dose-response curves*</u>

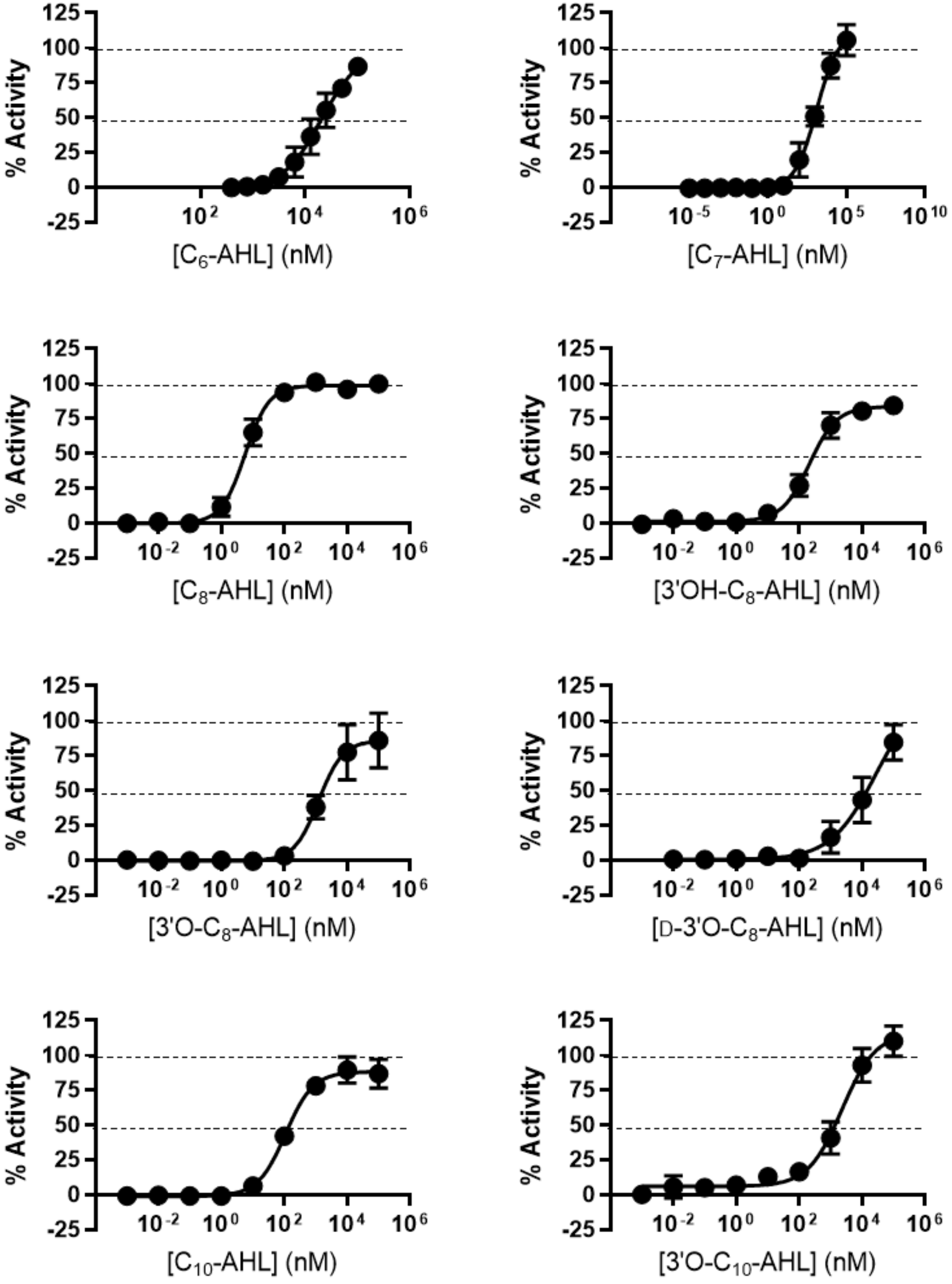

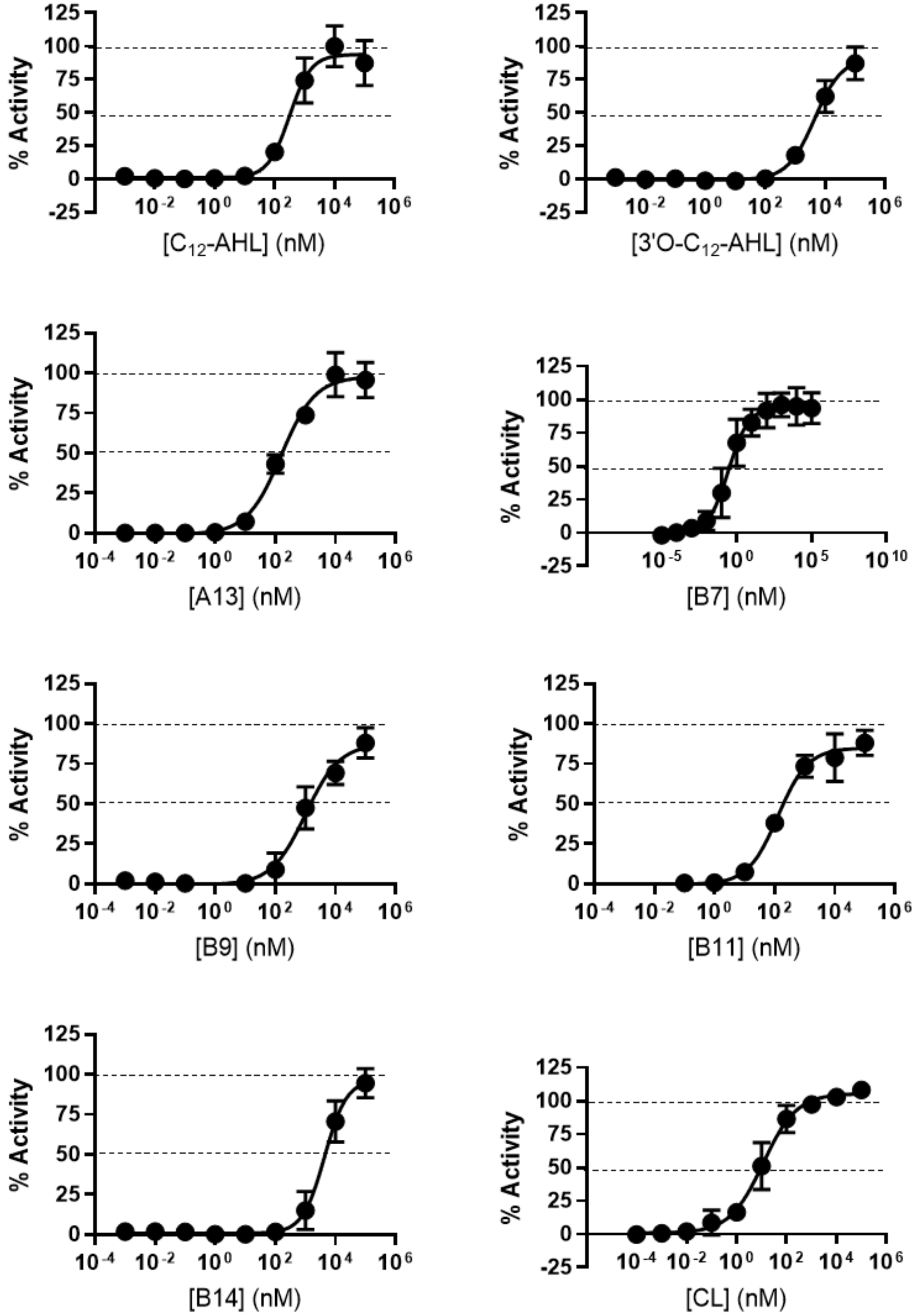
% Activity
[C$_{12}$-AHL] (nM)
[3'O-C$_{12}$-AHL] (nM)
[A13] (nM)
[B7] (nM)
[B9] (nM)
[B11] (nM)
[B14] (nM)
[CL] (nM)

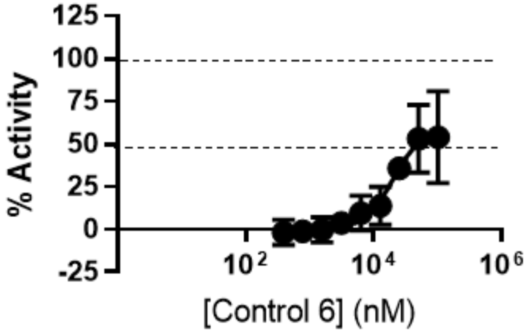
% Activity
125
100
75
50
25
0
-25
10^2
10^4
10^6
[Control 6] (nM)

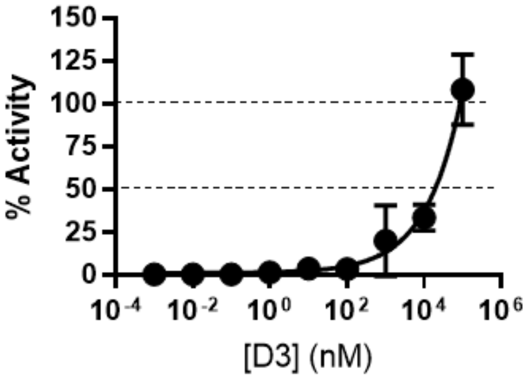
% Activity
150
125
100
75
50
25
0
10^{-4}
10^{-2}
10^0
10^2
10^4
10^6
[D3] (nM)

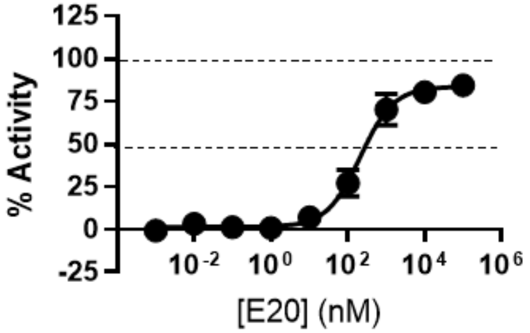
% Activity
125
100
75
50
25
0
-25
10^{-2}
10^0
10^2
10^4
10^6
[E20] (nM)

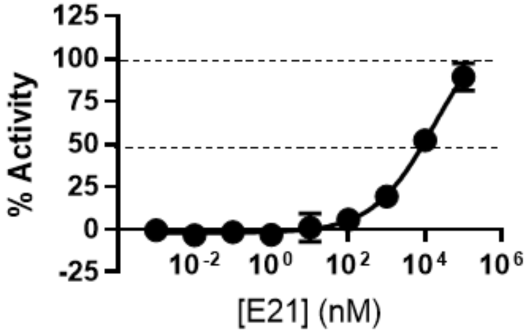
% Activity
125
100
75
50
25
0
-25
10^{-2}
10^0
10^2
10^4
10^6
[E21] (nM)

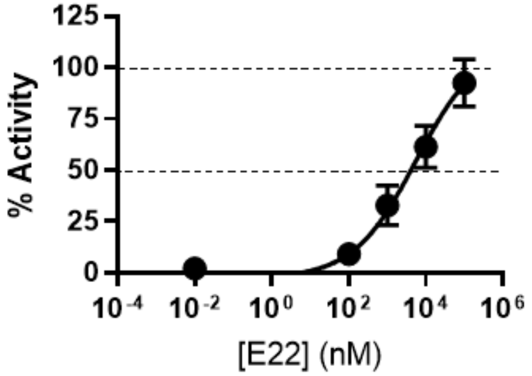
% Activity
125
100
75
50
25
0
10^{-4}
10^{-2}
10^0
10^2
10^4
10^6
[E22] (nM)

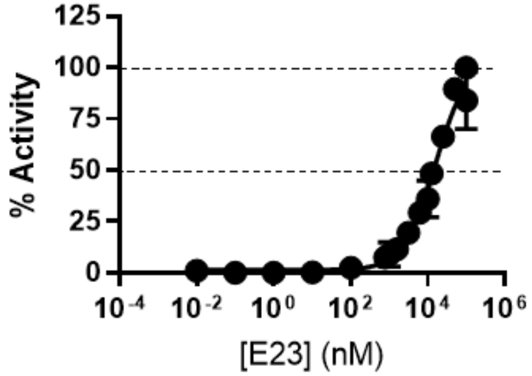
% Activity
125
100
75
50
25
0
10^{-4}
10^{-2}
10^0
10^2
10^4
10^6
[E23] (nM)

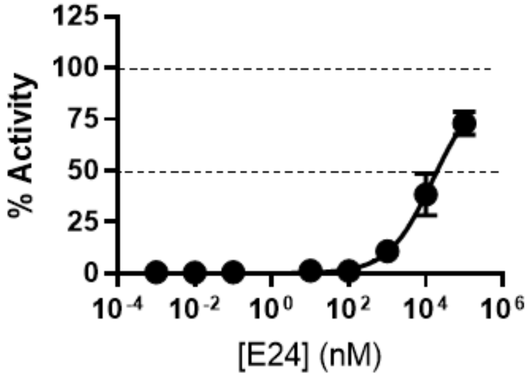
% Activity
125
100
75
50
25
0
10^{-4}
10^{-2}
10^0
10^2
10^4
10^6
[E24] (nM)

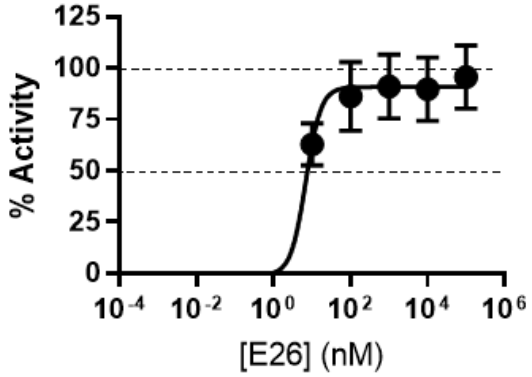
% Activity
125
100
75
50
25
0
10^{-4}
10^{-2}
10^0
10^2
10^4
10^6
[E26] (nM)

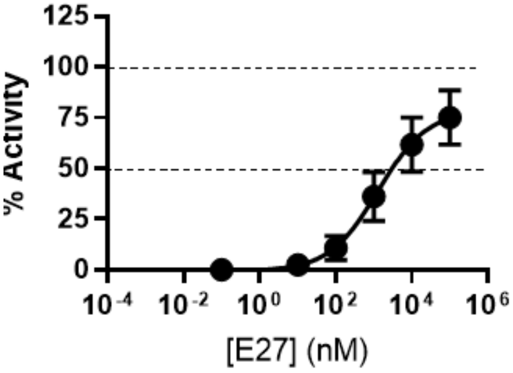
125
100
75
50
25
0
% Activity
10^{-4} 10^{-2} 10^{0} 10^{2} 10^{4} 10^{6}
[E27] (nM)

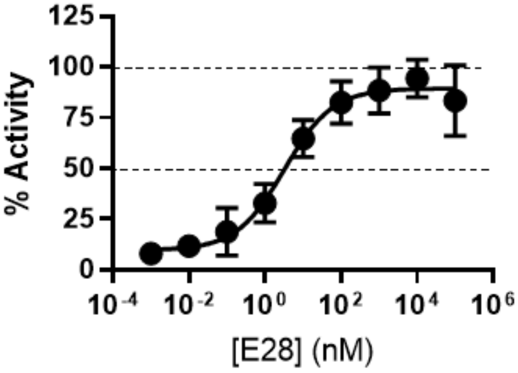
125
100
75
50
25
0
% Activity
10^{-4} 10^{-2} 10^{0} 10^{2} 10^{4} 10^{6}
[E28] (nM)

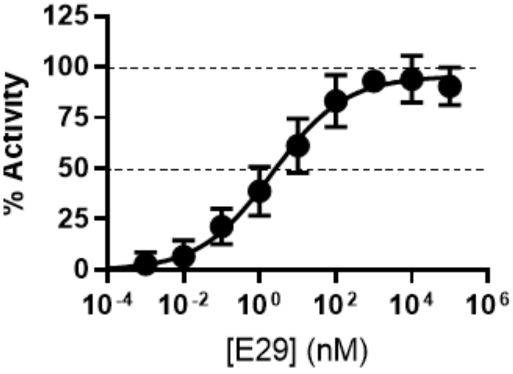
125
100
75
50
25
0
% Activity
10^{-4} 10^{-2} 10^{0} 10^{2} 10^{4} 10^{6}
[E29] (nM)

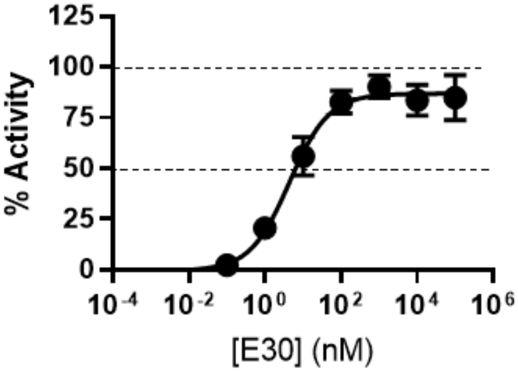
125
100
75
50
25
0
% Activity
10^{-4} 10^{-2} 10^{0} 10^{2} 10^{4} 10^{6}
[E30] (nM)

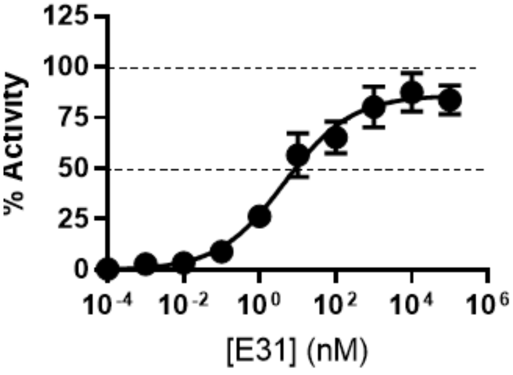
125
100
75
50
25
0
% Activity
10^{-4} 10^{-2} 10^{0} 10^{2} 10^{4} 10^{6}
[E31] (nM)

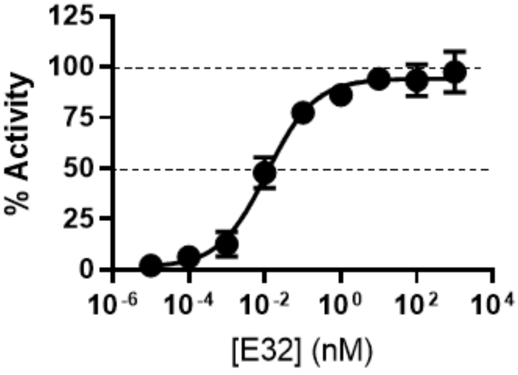
125
100
75
50
25
0
% Activity
10^{-6} 10^{-4} 10^{-2} 10^{0} 10^{2} 10^{4}
[E32] (nM)

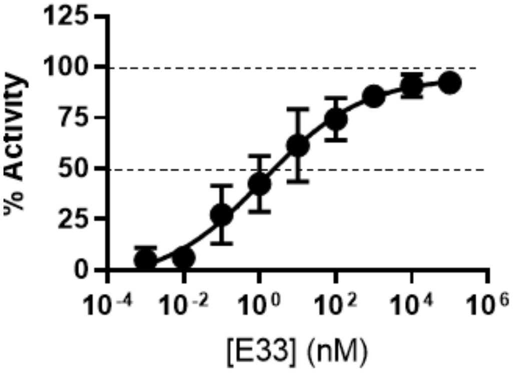
125
100
75
50
25
0
% Activity
10^{-4} 10^{-2} 10^{0} 10^{2} 10^{4} 10^{6}
[E33] (nM)

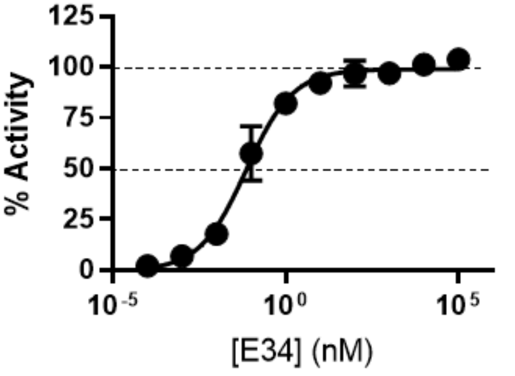
125
100
75
50
25
0
% Activity
10^{-5} 10^{0} 10^{5}
[E34] (nM)

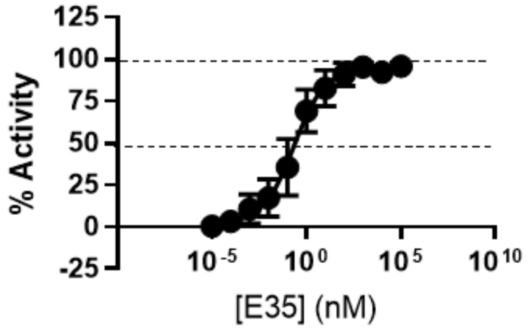
125
100
75
50
25
0
-25
% Activity
10^{-5}
10^{0}
10^{5}
10^{10}
[E35] (nM)

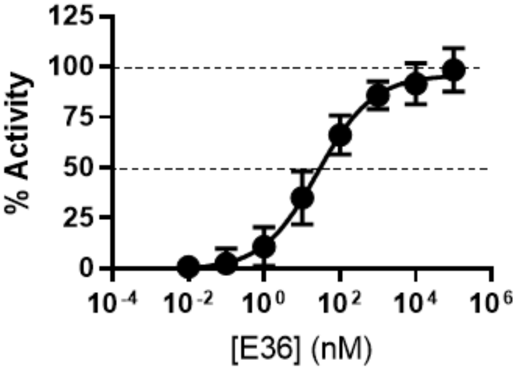
125
100
75
50
25
0
% Activity
10^{-4}
10^{-2}
10^{0}
10^{2}
10^{4}
10^{6}
[E36] (nM)

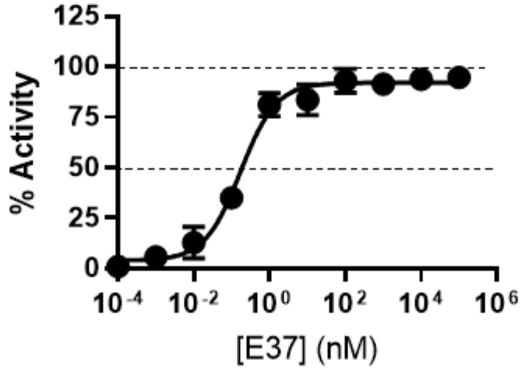
125
100
75
50
25
0
% Activity
10^{-4}
10^{-2}
10^{0}
10^{2}
10^{4}
10^{6}
[E37] (nM)

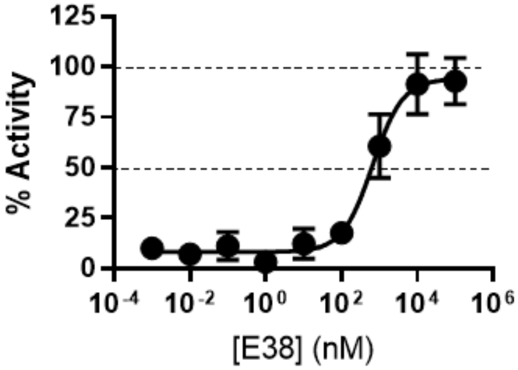
125
100
75
50
25
0
% Activity
10^{-4}
10^{-2}
10^{0}
10^{2}
10^{4}
10^{6}
[E38] (nM)

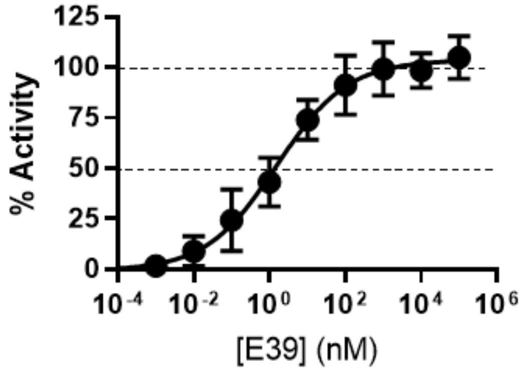
125
100
75
50
25
0
% Activity
10^{-4}
10^{-2}
10^{0}
10^{2}
10^{4}
10^{6}
[E39] (nM)

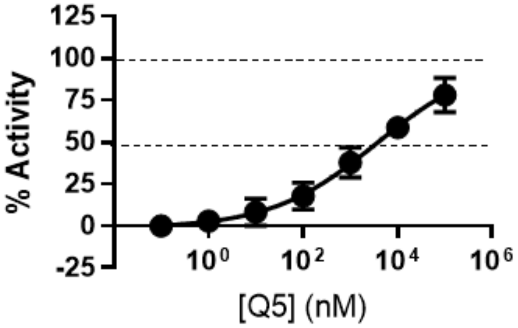
125
100
75
50
25
0
-25
% Activity
10^{0}
10^{2}
10^{4}
10^{6}
[Q5] (nM)

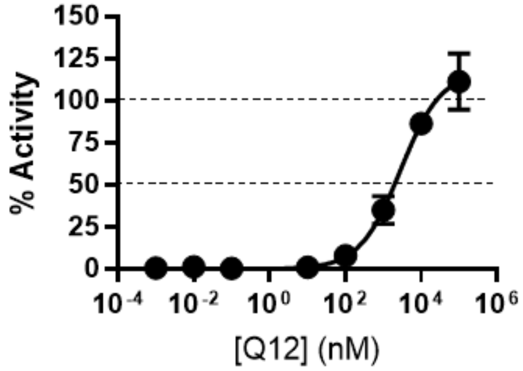
150
125
100
75
50
25
0
% Activity
10^{-4}
10^{-2}
10^{0}
10^{2}
10^{4}
10^{6}
[Q12] (nM)

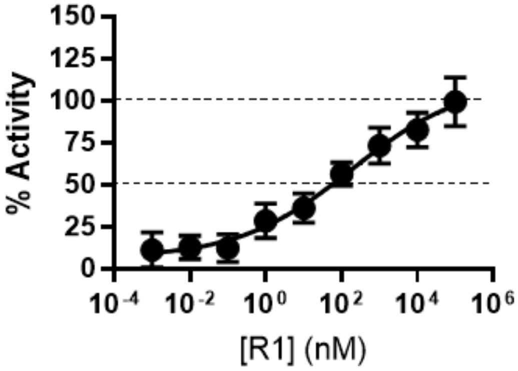
150
125
100
75
50
25
0
% Activity
10^{-4}
10^{-2}
10^{0}
10^{2}
10^{4}
10^{6}
[R1] (nM)

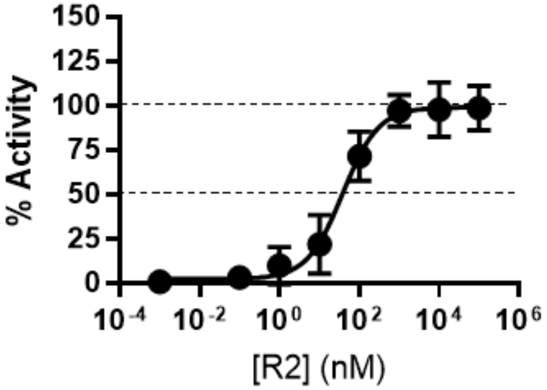
% Activity
150
125
100
75
50
25
0
10^{-4} 10^{-2} 10^{0} 10^{2} 10^{4} 10^{6}
[R2] (nM)

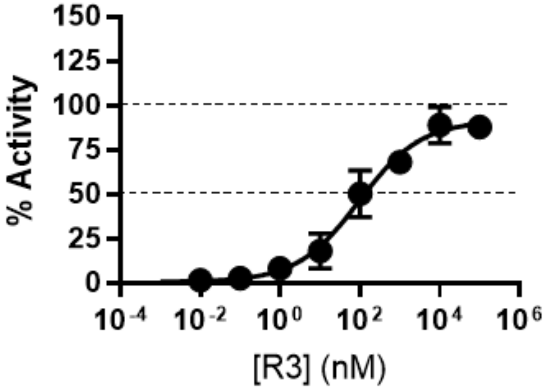
% Activity
150
125
100
75
50
25
0
10^{-4} 10^{-2} 10^{0} 10^{2} 10^{4} 10^{6}
[R3] (nM)

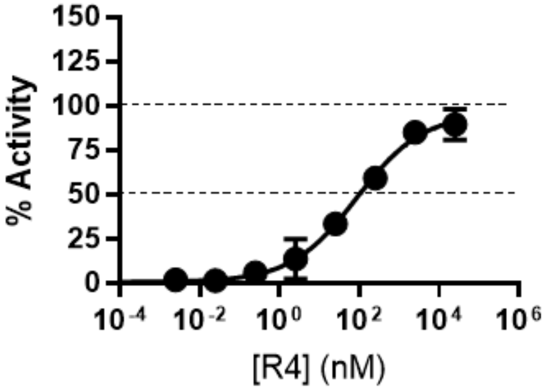
% Activity
150
125
100
75
50
25
0
10^{-4} 10^{-2} 10^{0} 10^{2} 10^{4} 10^{6}
[R4] (nM)

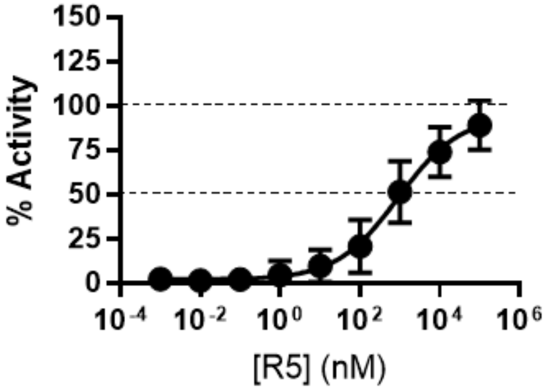
% Activity
150
125
100
75
50
25
0
10^{-4} 10^{-2} 10^{0} 10^{2} 10^{4} 10^{6}
[R5] (nM)

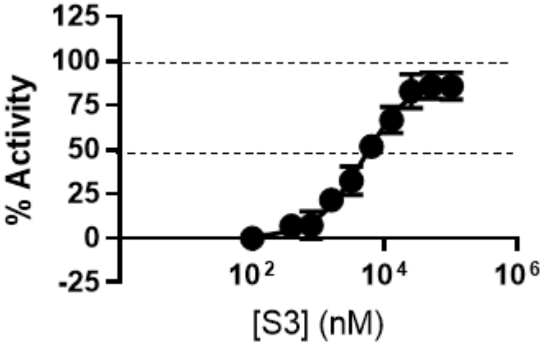
% Activity
125
100
75
50
25
0
-25
10^{2} 10^{4} 10^{6}
[S3] (nM)

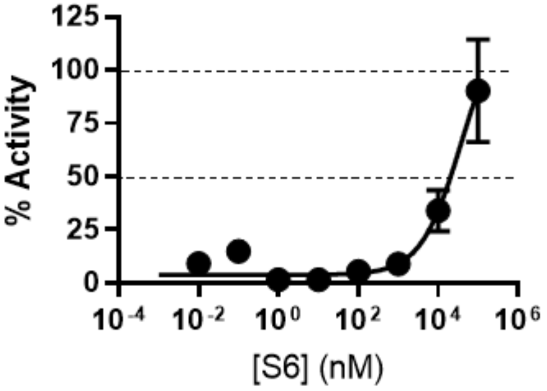
% Activity
125
100
75
50
25
0
10^{-4} 10^{-2} 10^{0} 10^{2} 10^{4} 10^{6}
[S6] (nM)

Ec-reporter CepR antagonist dose-response curves

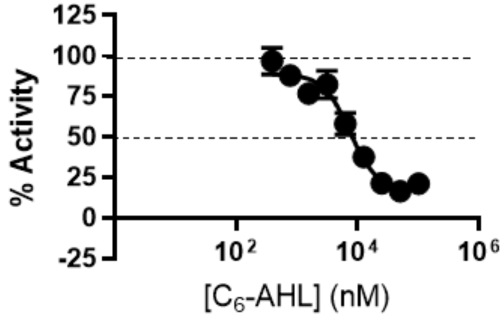

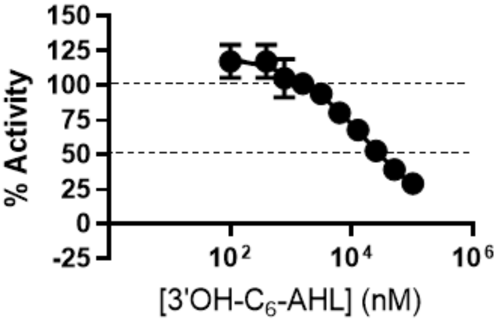

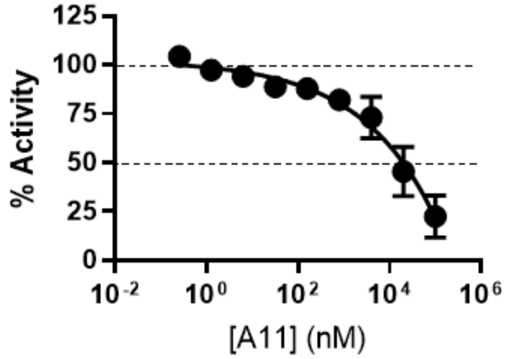

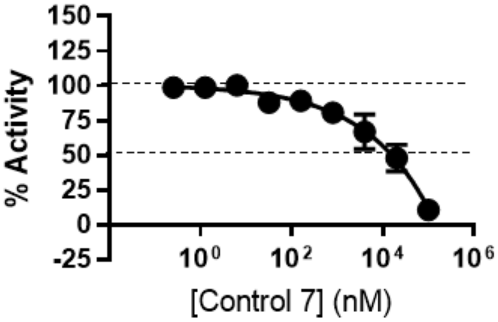

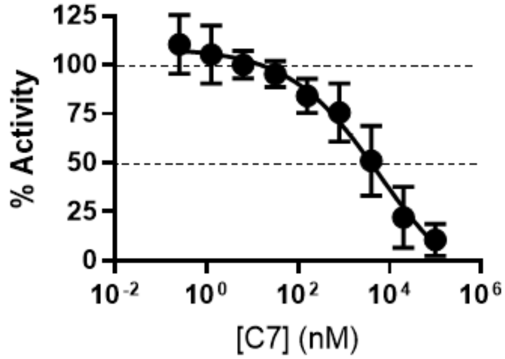

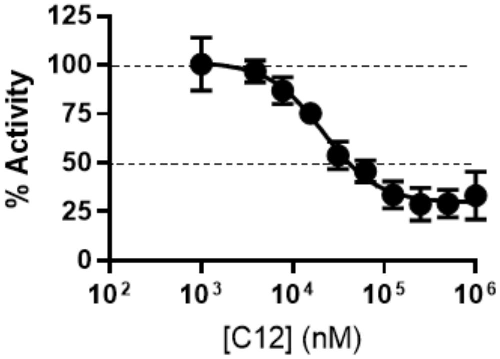

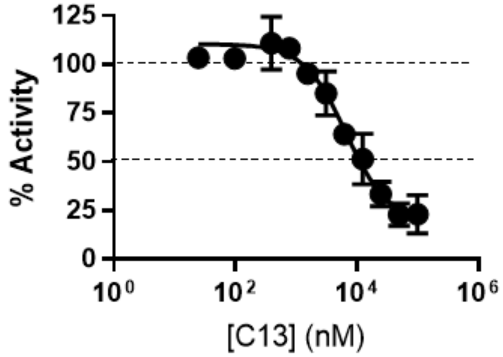

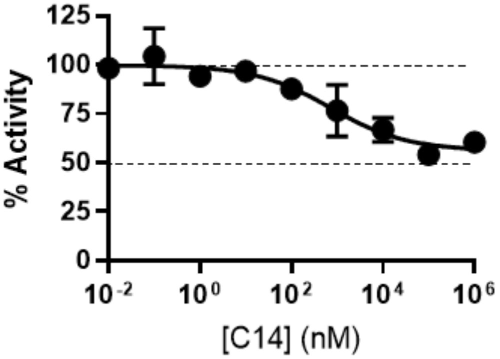

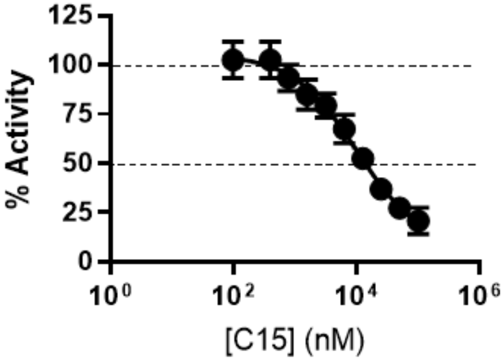
125
100
75
50
25
0
% Activity
10^0
10^2
10^4
10^6
[C15] (nM)

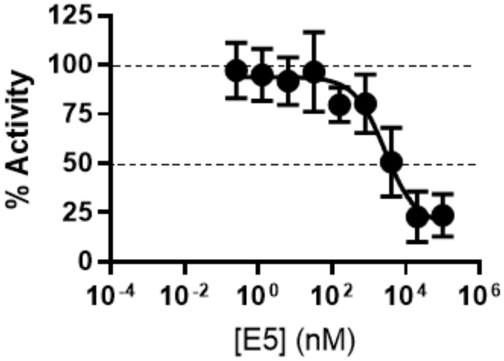
125
100
75
50
25
0
% Activity
10^-4
10^-2
10^0
10^2
10^4
10^6
[E5] (nM)

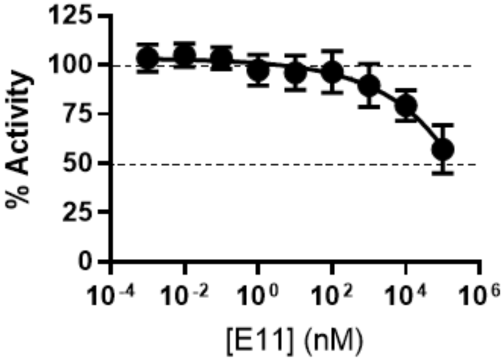
125
100
75
50
25
0
% Activity
10^-4
10^-2
10^0
10^2
10^4
10^6
[E11] (nM)

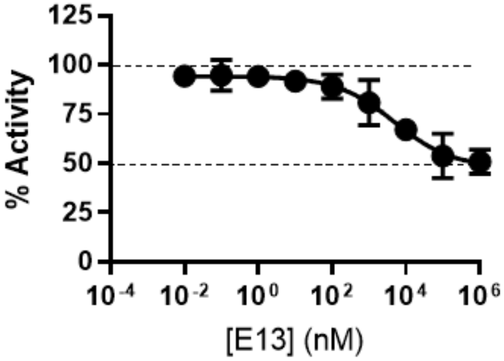
125
100
75
50
25
0
% Activity
10^-4
10^-2
10^0
10^2
10^4
10^6
[E13] (nM)

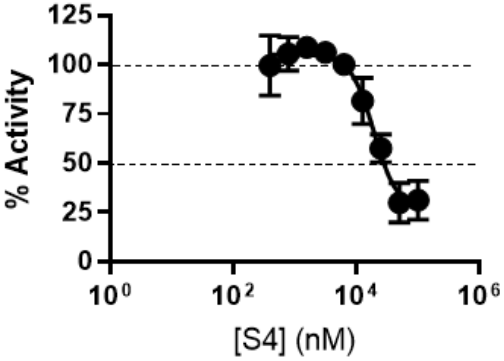
125
100
75
50
25
0
% Activity
10^0
10^2
10^4
10^6
[S4] (nM)

Ec-reporter CepR antagonist dose-response curves for compounds displaying upturn dose-response behavior

CepR antagonism dose-response curves for AHLs **C4-AHL**, **A12**, **B12**, **C6**, **C8**, **C9**, **E3**, **E7**, **E10**, and **E25** in the Ec-reporter are provided below. Two curves are shown for each AHL; the left curve incorporates all data points measured (showing CepR inhibition at lower concentrations and then inversion to CepR activation at higher concentrations), while the right curve incorporates only the data points where CepR inhibition was noted. The right curves were used to calculate IC_{50} and maximum inhibition values for each compound. Investigating the origins of this non-monotonic behavior is currently ongoing.

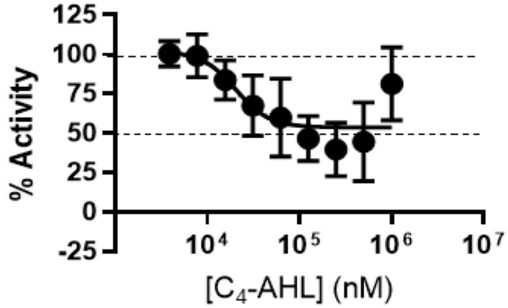

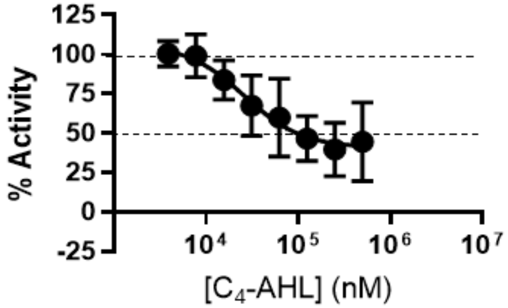

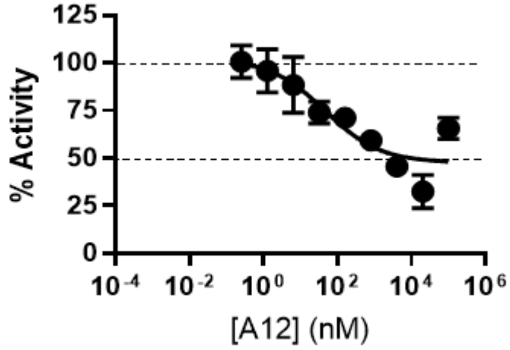

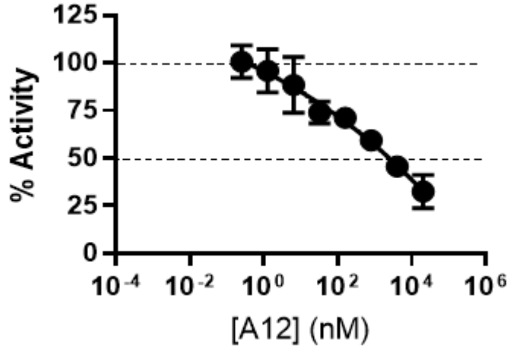

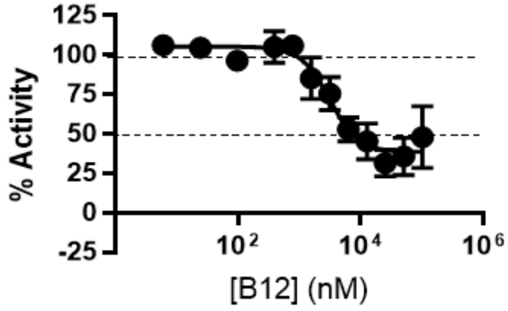

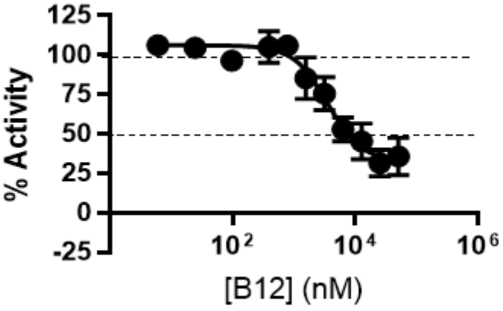

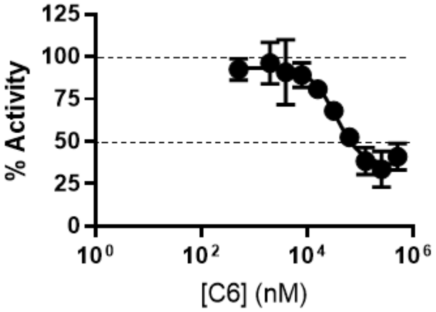
125
100
75
50
25
0
% Activity
10^0
10^2
10^4
10^6
[C6] (nM)

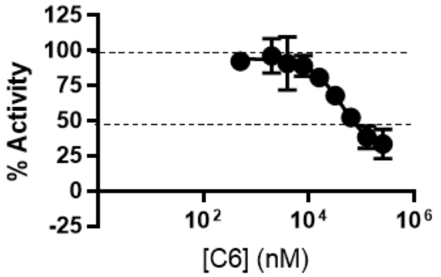
125
100
75
50
25
0
-25
% Activity
10^2
10^4
10^6
[C6] (nM)

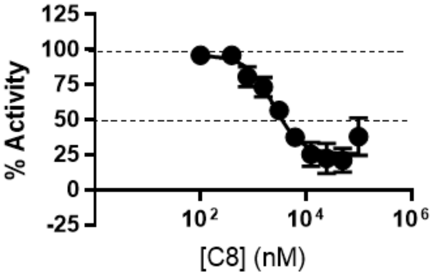
125
100
75
50
25
0
-25
% Activity
10^2
10^4
10^6
[C8] (nM)

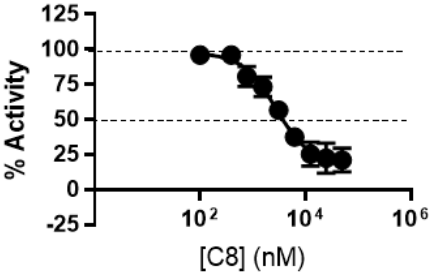
125
100
75
50
25
0
-25
% Activity
10^2
10^4
10^6
[C8] (nM)

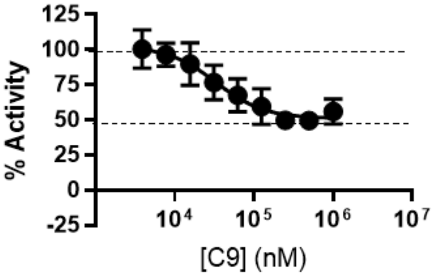
125
100
75
50
25
0
-25
% Activity
10^4
10^5
10^6
10^7
[C9] (nM)

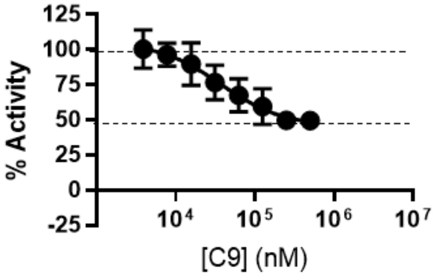
125
100
75
50
25
0
-25
% Activity
10^4
10^5
10^6
10^7
[C9] (nM)

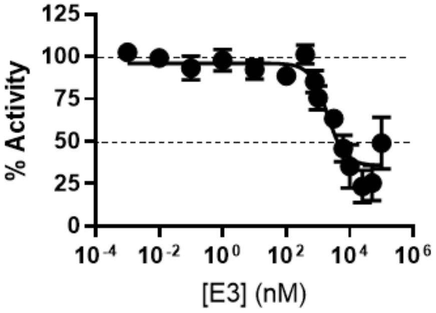
125
100
75
50
25
0
% Activity
10^-4
10^-2
10^0
10^2
10^4
10^6
[E3] (nM)

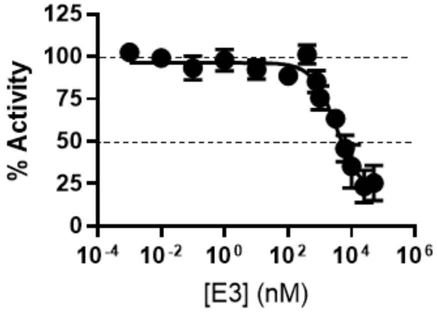
125
100
75
50
25
0
% Activity
10^-4
10^-2
10^0
10^2
10^4
10^6
[E3] (nM)

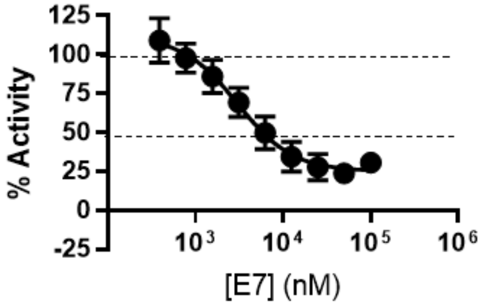
% Activity
[E7] (nM)

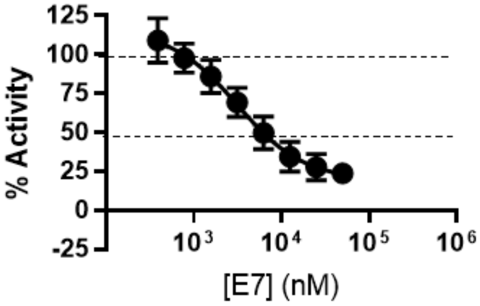
% Activity
[E7] (nM)

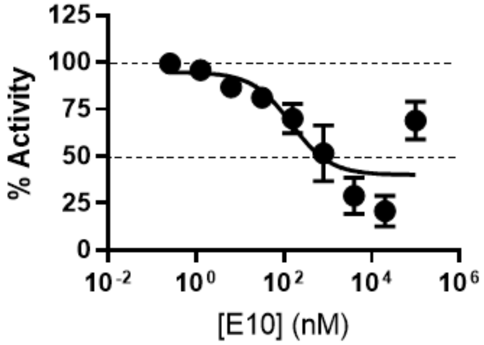
% Activity
[E10] (nM)

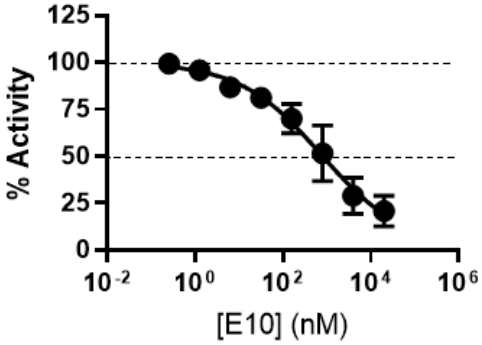
% Activity
[E10] (nM)

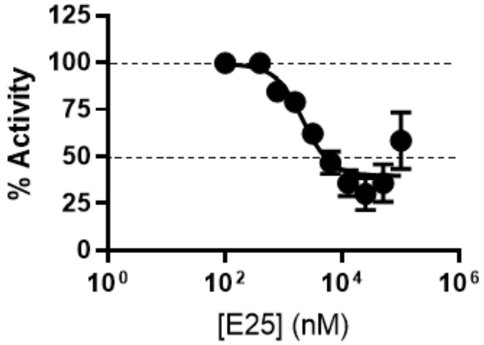
% Activity
[E25] (nM)

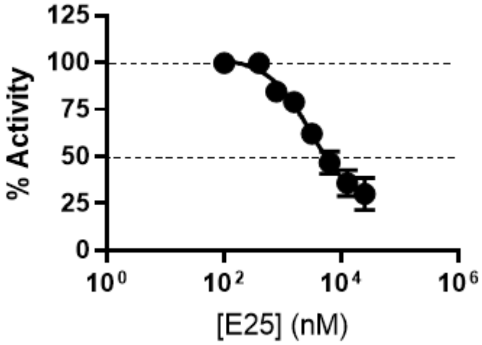
% Activity
[E25] (nM)

Bm-reporter CepR agonist dose-response curves

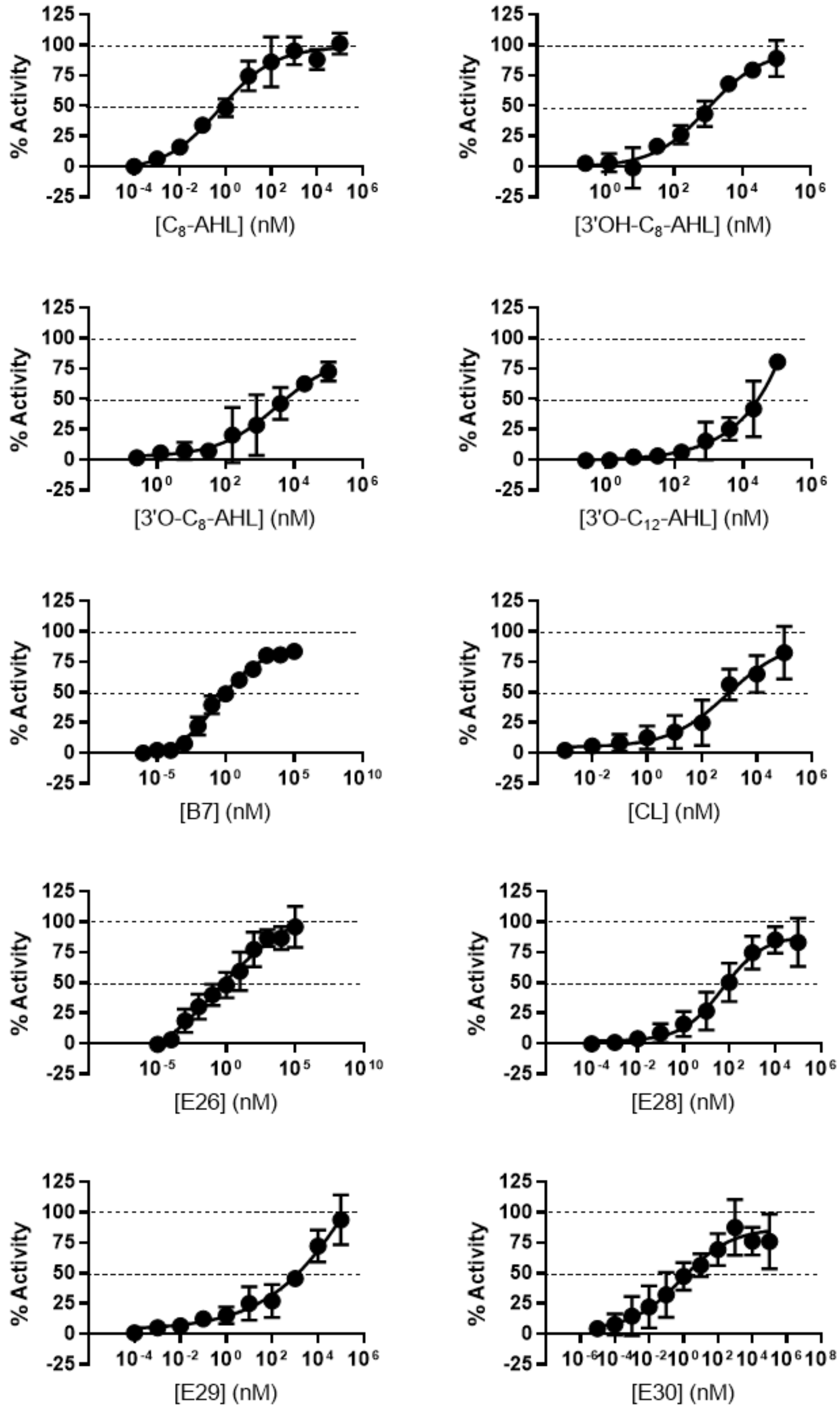

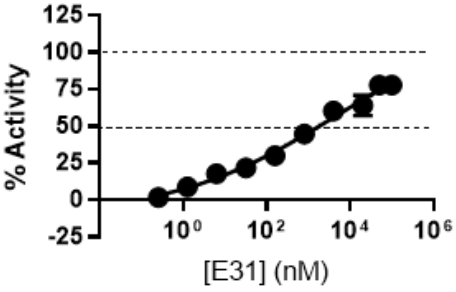
% Activity
[E31] (nM)

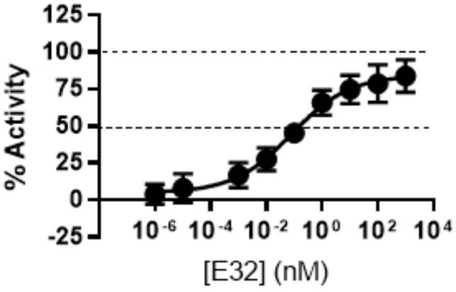
% Activity
[E32] (nM)

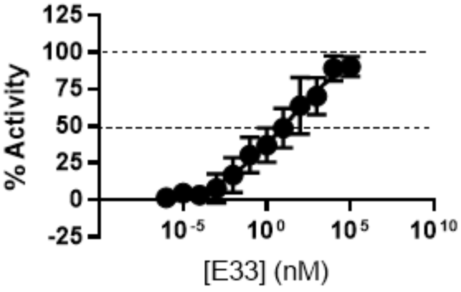
% Activity
[E33] (nM)

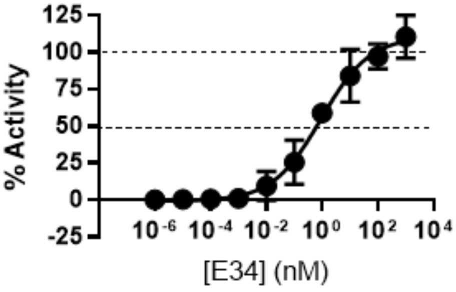
% Activity
[E34] (nM)

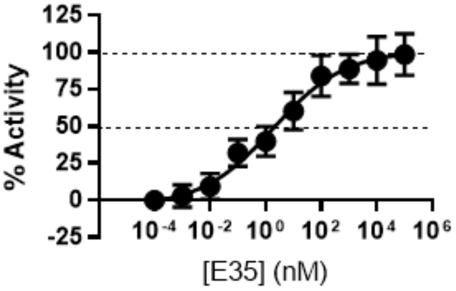
% Activity
[E35] (nM)

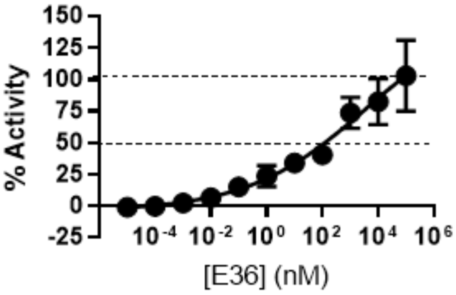
% Activity
[E36] (nM)

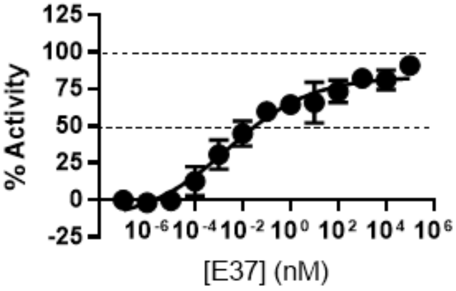
% Activity
[E37] (nM)

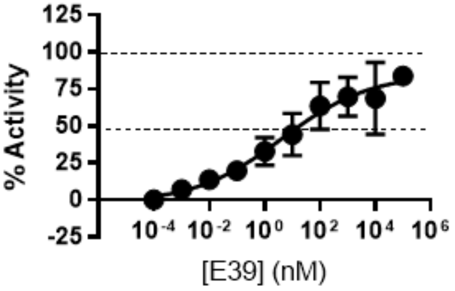
% Activity
[E39] (nM)

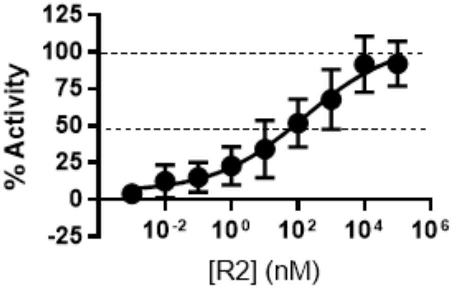
% Activity
[R2] (nM)

Bm-reporter CepR antagonist dose-response curves

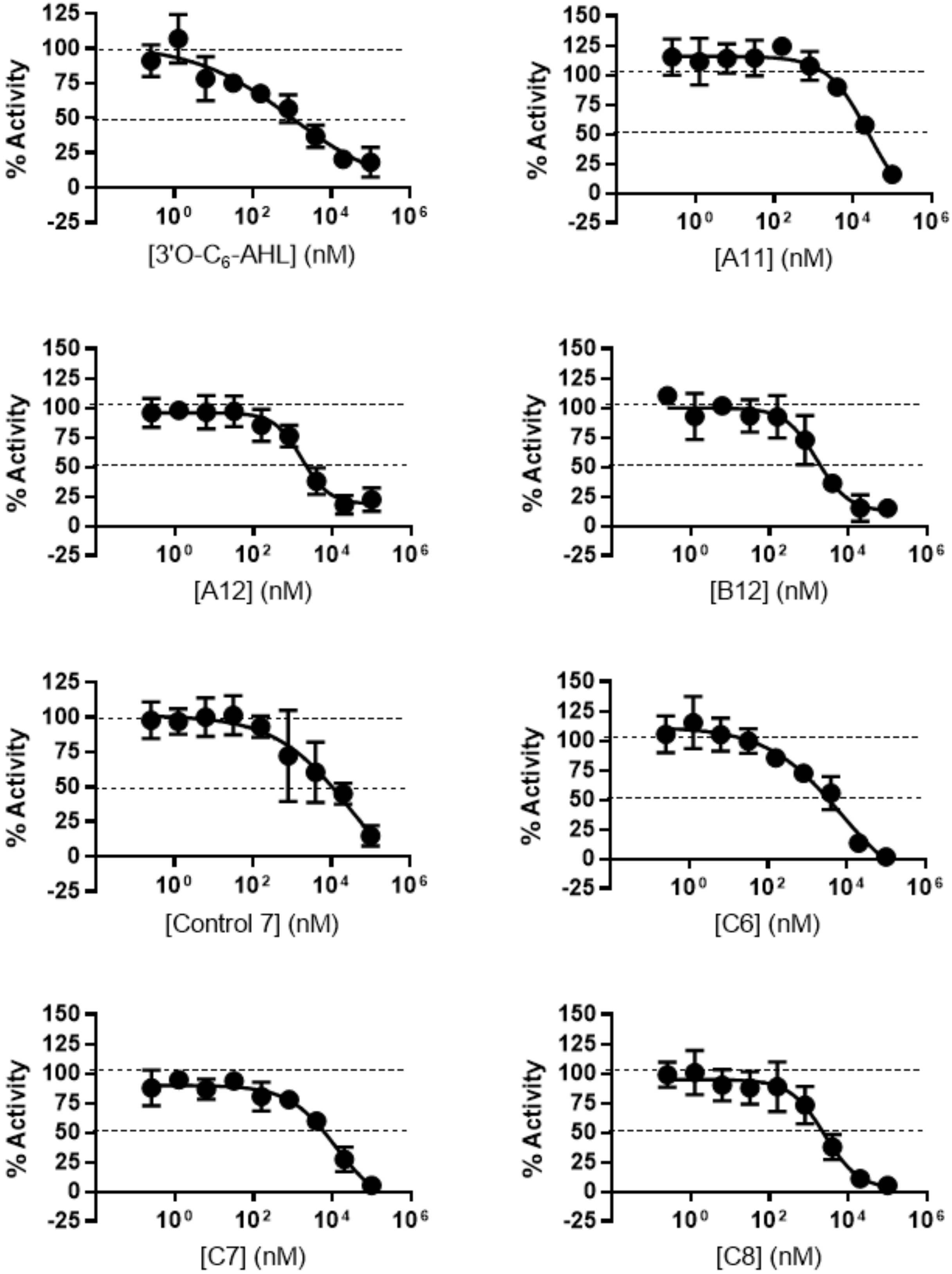

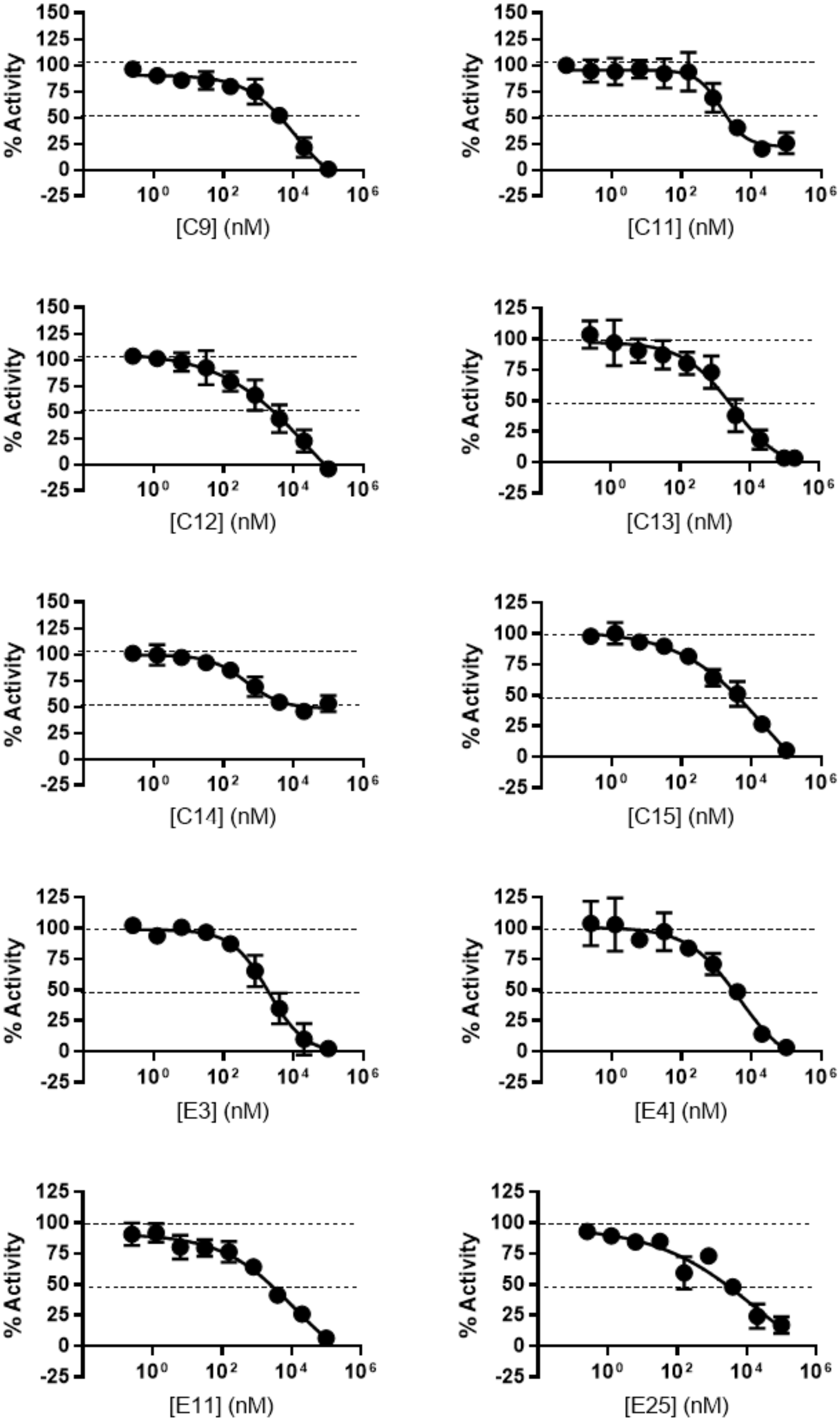

% Activity
[C9] (nM)
[C11] (nM)
[C12] (nM)
[C13] (nM)
[C14] (nM)
[C15] (nM)
[E3] (nM)
[E4] (nM)
[E11] (nM)
[E25] (nM)

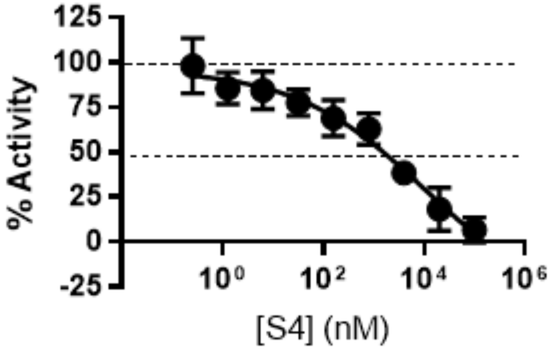

125
100
75
50
25
0
-25
% Activity
10⁰
10²
10⁴
10⁶
[S4] (nM)

Bm-reporter CepR antagonist dose-response curves for compound displaying upturn dose-response behavior

CepR antagonism dose-response curves for **C_4-AHL** in the Bm-reporter are provided below. Two curves are shown; the left curve incorporates all data points measured (showing CepR inhibition at lower concentrations and then inversion to CepR activation at higher concentrations), while the right curve incorporates only the data points where CepR inhibition was noted. The right curve was used to calculate IC_{50} and maximum inhibition value for **C_4-AHL**. Again, investigating the origins of this non-monotonic behavior is currently ongoing.

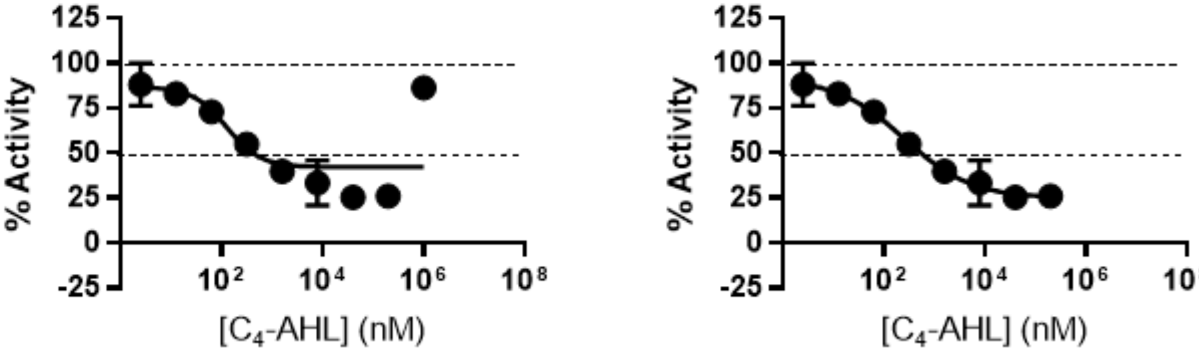

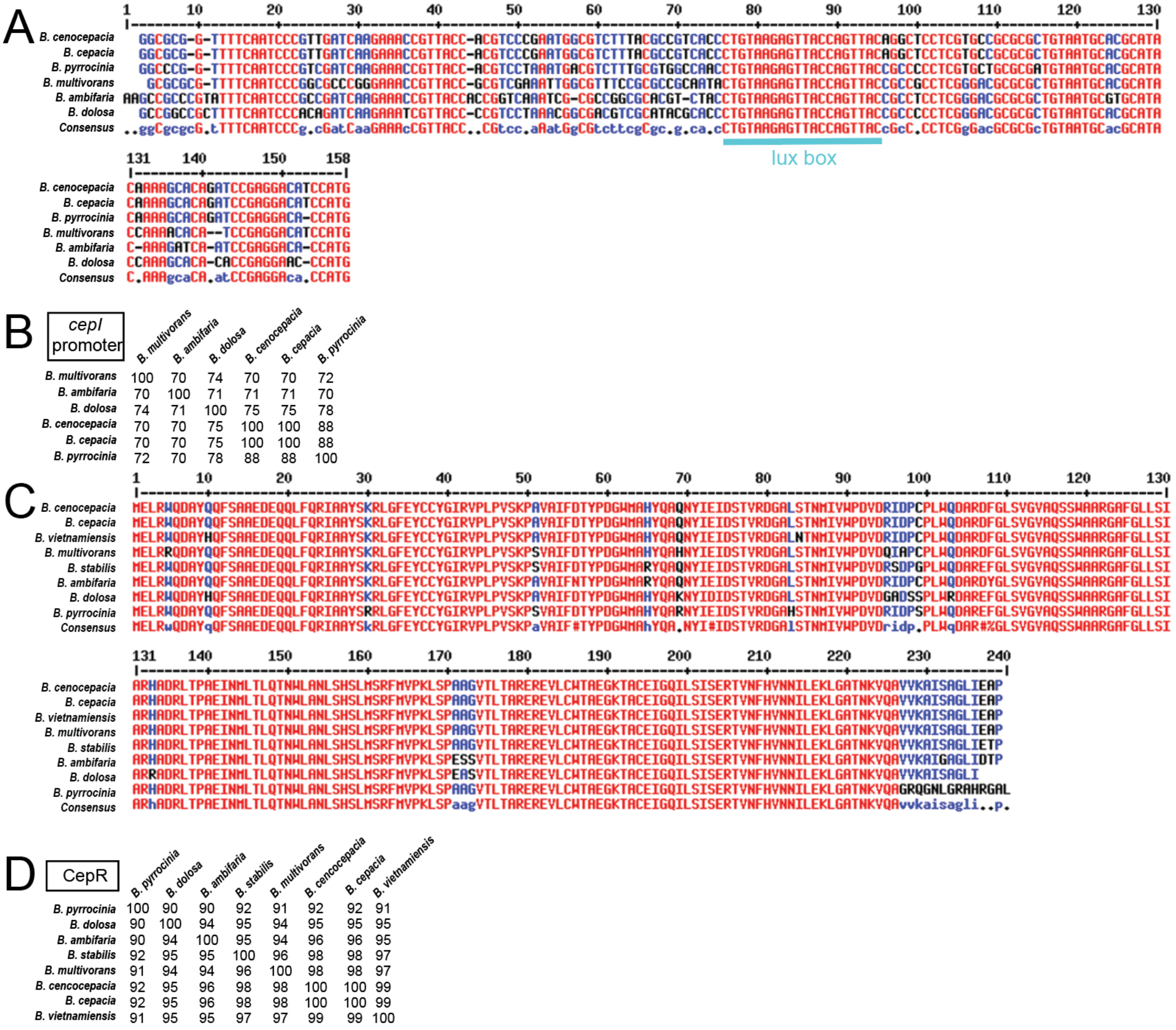

Figure S3. *cepI* promoter and CepR conservation analysis. (A) Alignment of the *cepI* promoter region (from -150 through the start codon) among Bcc member species. Red indicates high (≥ 90%), blue indicates medium (50–90%), and black indicates low (≤ 50%) conservation. (B) The percent identity matrix for the *cepI* promoter region. (C) Alignment of the CepR protein coding sequence among Bcc member species. Red indicates high (≥ 90%), blue indicates medium (50–90%), and black indicates low (≤ 50%) conservation. (D) The percent identity matrix for the CepR protein. Alignments were generated using MultAlign.[14] Clustal Omega was used to determine the percent identity matrix.[15]

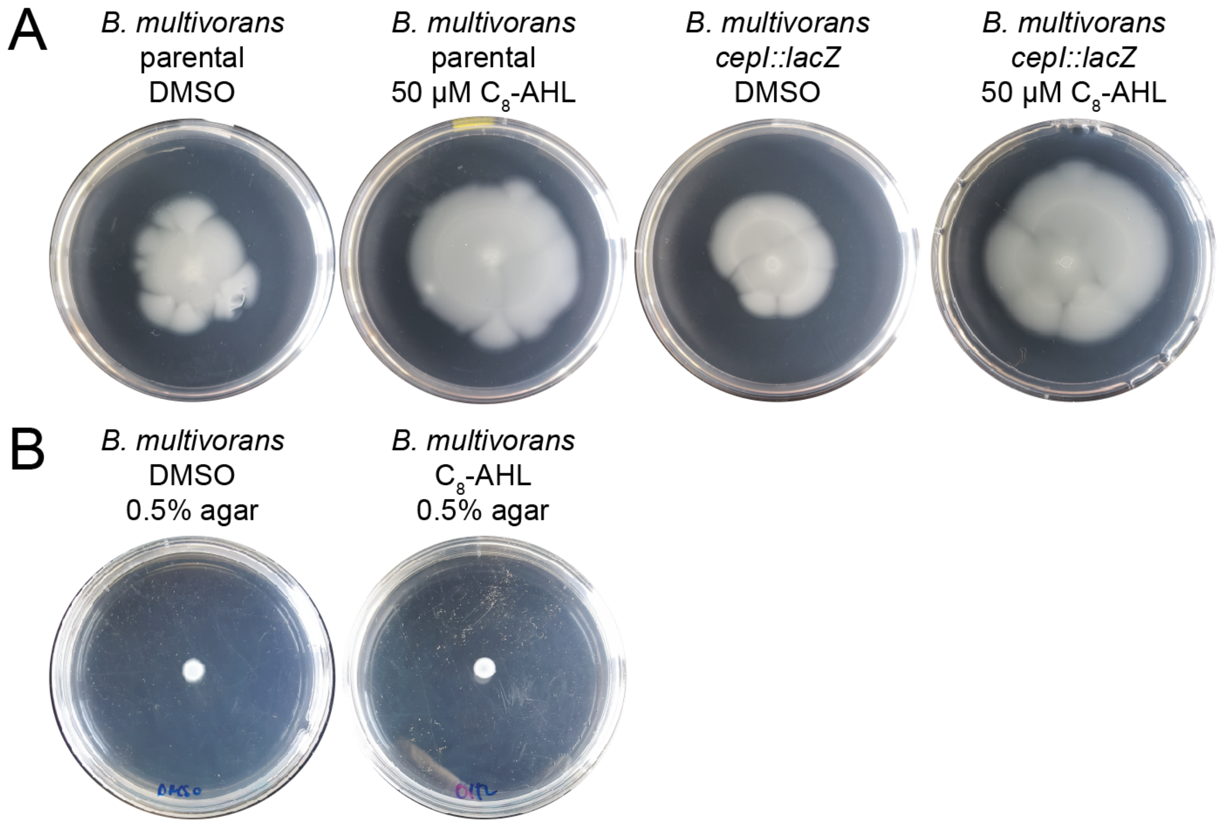

Figure S4. Motility assays (A) on 0.3% agar (i.e., to measure swimming) with the *B. multivorans* parental and *cepI::lacZ* organisms, using +/- 50 µM C_8-AHL and (B) on 0.5% agar (i.e., to measure swarming) with the *B. multivorans* parental organism, using +/- 50 µM C_8-AHL. See main text for assay methods.

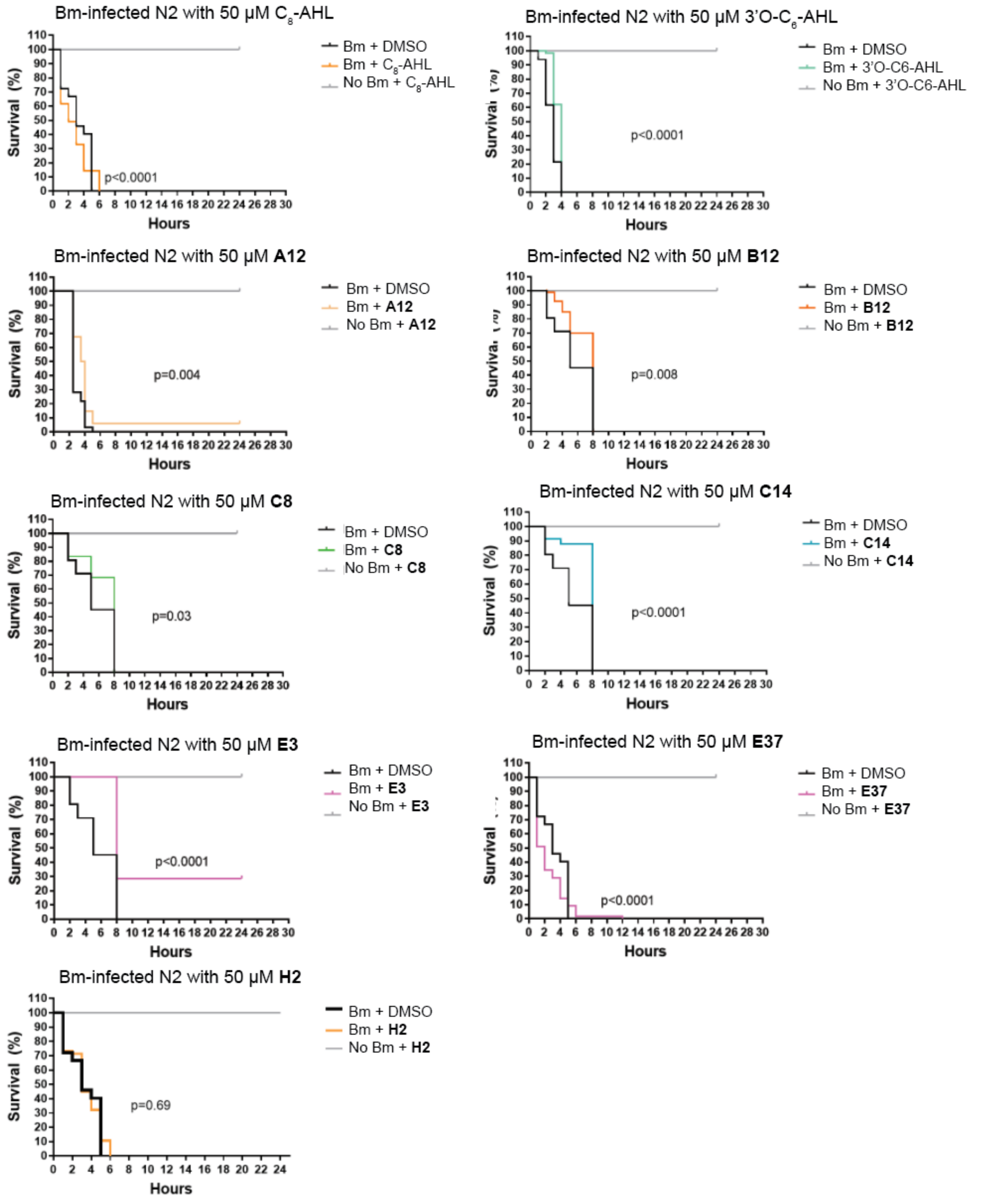

Figure S5. *C. elegans* survival assays with *B. multivorans* and lead compounds in this study or DMSO. See main text for assay methods.

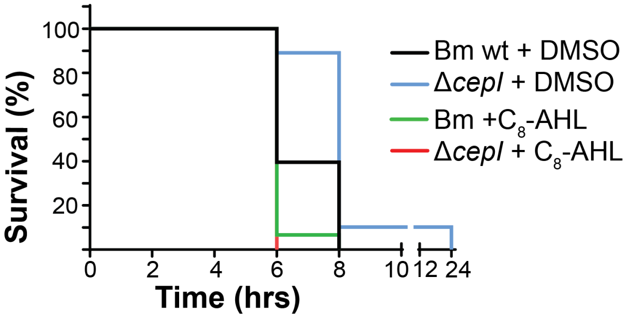

Figure S6. *C. elegans* survival assays with *B. multivorans* and *B. multivorans cepI::lacZ*, using +/- C_8-AHL or DMSO. See main text for assay methods.

Table S6. Strains and plasmids used in this study.

**Strain or plasmid**	**Description**	**Reference or source**
Plasmid:		
Burkholderia multivorans ATCC17616	Wild type strain	ATCC
Burkholderia multivorans cepI::lacZ	ATCC17616 with *cepI::lacZ*	This study
Escherichia coli DH5α	Cloning strain	Invitrogen
Caenorhabditis elegans N2	Survival assays	CGC – University of Minnesota
Escherichia coli RHO3	Donor strain	[16]
Escherichia coli XL-1 Blue	Cloning strain	Thermo Fisher Scientific
Escherichia coli JLD271	K-12 ΔlacX74 sdiA271::Cam; Cam^R	[9]
Escherichia coli OP50	*C. elegans* food source	
Plasmid:		
pEXG2	Suicide plasmid	[17]
pUC18-miniTn7T-Gm-lacZ	Source of *lacZ*	[18]
pEXG2 Δ*cepI*	pEXG2 with Δ*cepI*	This study
pEXG2 P*cepI::lacZ*	pEXG2 Δ*cepI* with *lacZ* inserted into XbaI and ScaI sites	This study
pSC11-*cepI**	Broad host range *cepI'-lacZ* reporter plasmid; AmpR	This study, derived from [19]
pJN105-*cepR*	CepR expression vector with pBAD promoter, pBBRMCS backbone; GentR	This study, derived from [20]
DNA:		
Burkholderia cenocepacia genomic DNA	BAA-245D-5	ATCC
9-SalI-cepI*-F	CATGTCGACCAACCGGCCGCGCATTCCTCTGAC	This study
10-BamHI-cepI'-R	CATGGATCCCCCTTCCTCGTGAACGAAGGTCTGCAT	This study
7-EcoRI-cepR-F	CATGAATTCATGGAACTGCGCTGGCAGGATGCCTAC	This study
8-XbaI-cepR-R	CATTCTAGATCAGGGTGCTTCGATGAGCCCGGC	This study

11-lacZ-R2	CCAGTGAATTCCCTTTCATTATTAATACCCTC	This study
2-pBADprom-F	GTTTCTCCATACCCGTTTTTTTGGGCTAGC	This study
13-cepR-R2	GTACGCGAATGCCGTAACAGCAGTATT	This study
14-cepR-F2	CGTTCGGGCTGCTAAGCATCGCC	This study

Table footnote: Cam^R, chloramphenicol resistance; Amp^R, Ampicillin resistance; $Gent^R$, gentamicin resistance.

References

1 Moore, J. D., Rossi, F. M., Welsh, M. A., Nyffeler, K. E. & Blackwell, H. E. A Comparative Analysis of Synthetic Quorum Sensing Modulators in *Pseudomonas aeruginosa*: New Insights into Mechanism, Active Efflux Susceptibility, Phenotypic Response, and Next-Generation Ligand Design. *J Am Chem Soc* **137**, 14626-14639, doi:10.1021/jacs.5b06728 (2015).

2 Geske, G. D., O'Neill, J. C., Miller, D. M., Mattmann, M. E. & Blackwell, H. E. Modulation of bacterial quorum sensing with synthetic ligands: systematic evaluation of *N*-acylated homoserine lactones in multiple species and new insights into their mechanisms of action. *J Am Chem Soc* **129**, 13613-13625, doi:10.1021/ja074135h (2007).

3 Stacy, D. M., Welsh, M. A., Rather, P. N. & Blackwell, H. E. Attenuation of quorum sensing in the pathogen *Acinetobacter baumannii* using non-native *N*-acyl homoserine lactones. *ACS Chem Biol* **7**, 1719-1728, doi:10.1021/cb300351x (2012).

4 Geske, G. D., Mattmann, M. E. & Blackwell, H. E. Evaluation of a focused library of *N*-aryl L-homoserine lactones reveals a new set of potent quorum sensing modulators. *Bioorg Med Chem Lett* **18**, 5978-5981, doi:10.1016/j.bmcl.2008.07.089 (2008).

5 McInnis, C. E. & Blackwell, H. E. Thiolactone modulators of quorum sensing revealed through library design and screening. *Bioorg Med Chem* **19**, 4820-4828, doi:10.1016/j.bmc.2011.06.071 (2011).

6 Gerdt, J. P. *et al.* Chemical Interrogation of LuxR-type Quorum Sensing Receptors Reveals New Insights into Receptor Selectivity and the Potential for Interspecies Bacterial Signaling. *ACS Chem Biol* **12**, 2457-2464, doi:10.1021/acschembio.7b00458 (2017).

7 Mattmann, M. E., Shipway, P. M., Heth, N. J. & Blackwell, H. E. Potent and selective synthetic modulators of a quorum sensing repressor in *Pseudomonas aeruginosa* identified from second-generation libraries of *N*-acylated L-homoserine lactones. *Chembiochem* **12**, 942-949, doi:10.1002/cbic.201000708 (2011).

8 Geske, G. D., O'Neill, J. C. & Blackwell, H. E. *N*-phenylacetanoyl-L-homoserine lactones can strongly antagonize or superagonize quorum sensing in *Vibrio fischeri*. *ACS Chem Biol* **2**, 315-319, doi:10.1021/cb700036x (2007).

9 Swem, L. R. *et al.* A quorum-sensing antagonist targets both membrane-bound and cytoplasmic receptors and controls bacterial pathogenicity. *Mol Cell* **35**, 143-153, doi:10.1016/j.molcel.2009.05.029 (2009).

10 O'Loughlin, C. T. *et al.* A quorum-sensing inhibitor blocks *Pseudomonas aeruginosa* virulence and biofilm formation. *Proc Natl Acad Sci USA* **110**, 17981-17986, doi:10.1073/pnas.1316981110 (2013).

11 Morkunas, B. *et al.* Inhibition of the production of the *Pseudomonas aeruginosa* virulence factor pyocyanin in wild-type cells by quorum sensing autoinducer-mimics. *Org Biomol Chem* **10**, 8452-8464, doi:10.1039/c2ob26501j (2012).

12 Zakhari, J. S. *et al.* Synthesis and molecular modeling provide insight into a *Pseudomonas aeruginosa* quorum sensing conundrum. *J Am Chem Soc* **133**, 3840-3842, doi:10.1021/ja111138y (2011).

13 Gerdt, J. P., McInnis, C. E., Schell, T. L., Rossi, F. M. & Blackwell, H. E. Mutational analysis of the quorum-sensing receptor LasR reveals interactions that govern activation and inhibition by nonlactone ligands. *Chem Biol* **21**, 1361-1369, doi:10.1016/j.chembiol.2014.08.008 (2014).

14 Corpet, F. Multiple sequence alignment with hierarchical clustering. *Nucleic Acids Res* **16**, 10881-10890 (1988).

15 Sievers, F. *et al.* Fast, scalable generation of high-quality protein multiple sequence alignments using Clustal Omega. *Mol Syst Biol* **7**, 539, doi:10.1038/msb.2011.75 (2011).

16 Lopez, C. M., Rholl, D. A., Trunck, L. A. & Schweizer, H. P. Versatile dual-technology system for markerless allele replacement in *Burkholderia pseudomallei*. *Appl Environ Microbiol* **75**, 6496-6503, doi:10.1128/AEM.01669-09 (2009).
17 Rietsch, A., Vallet-Gely, I., Dove, S. L. & Mekalanos, J. J. ExsE, a secreted regulator of type III secretion genes in *Pseudomonas aeruginosa*. *Proc Natl Acad Sci USA* **102**, 8006-8011, doi:10.1073/pnas.0503005102 (2005).
18 Choi, K. H. & Schweizer, H. P. mini-Tn7 insertion in bacteria with single attTn7 sites: example *Pseudomonas aeruginosa*. *Nat Prot* **1**, 153-161, doi:10.1038/nprot.2006.24 (2006).
19 Chugani, S. A. *et al.* QscR, a modulator of quorum-sensing signal synthesis and virulence in *Pseudomonas aeruginosa*. *Proc Natl Acad Sci USA* **98**, 2752-2757, doi:10.1073/pnas.051624298 (2001).
20 Lee, J. H., Lequette, Y. & Greenberg, E. P. Activity of purified QscR, a *Pseudomonas aeruginosa* orphan quorum-sensing transcription factor. *Mol Microbiol* **59**, 602-609, doi:10.1111/j.1365-2958.2005.04960.x (2006).
